# Supplementary material for: A dual‐AAV approach restores fast exocytosis and partially rescues auditory function in deaf otoferlin knock‐out mice
Source: EMBO Mol Med. 2018 Dec 3;11(1):e9396. doi: 10.15252/emmm.201809396 (PMC6328916; doi:10.15252/emmm.201809396)
Supplement: Supplementary file 1 — Appendix [file EMMM-11-e9396-s001.pdf]

Appendix

for

A dual-AAV approach restores fast IHC exocytosis and partially rescues auditory function in deaf otoferlin knock-out mice

Hanan Al-Moyed[1,2], Andreia P. Cepeda[1,2], Sangyong Jung[3,4], Tobias Moser[2,3,4], Sebastian Kügler[5,*], Ellen Reisinger[1,*]

Table of content

Appendix Figure S1 - Contralateral non-injected *Otof*$^{-/-}$ ears do not show dual-AAV mediated eGFP and otoferlin expression

Appendix Figure S2 - OHCs and IHCs from contralateral non-injected *Otof*$^{-/-}$ ears do not show dual-AAV mediated eGFP and otoferlin expression

Appendix Figure S3 - OHCs were sparsely transduced with dual-AAV vectors.

Appendix Figure S4 - Otoferlin dual-AAV-TS injected and non-injected contralateral ears of wild-type mice did not differ in ABR thresholds and wave latencies.

Appendix Table S1 – Statistical tests and *P*-values

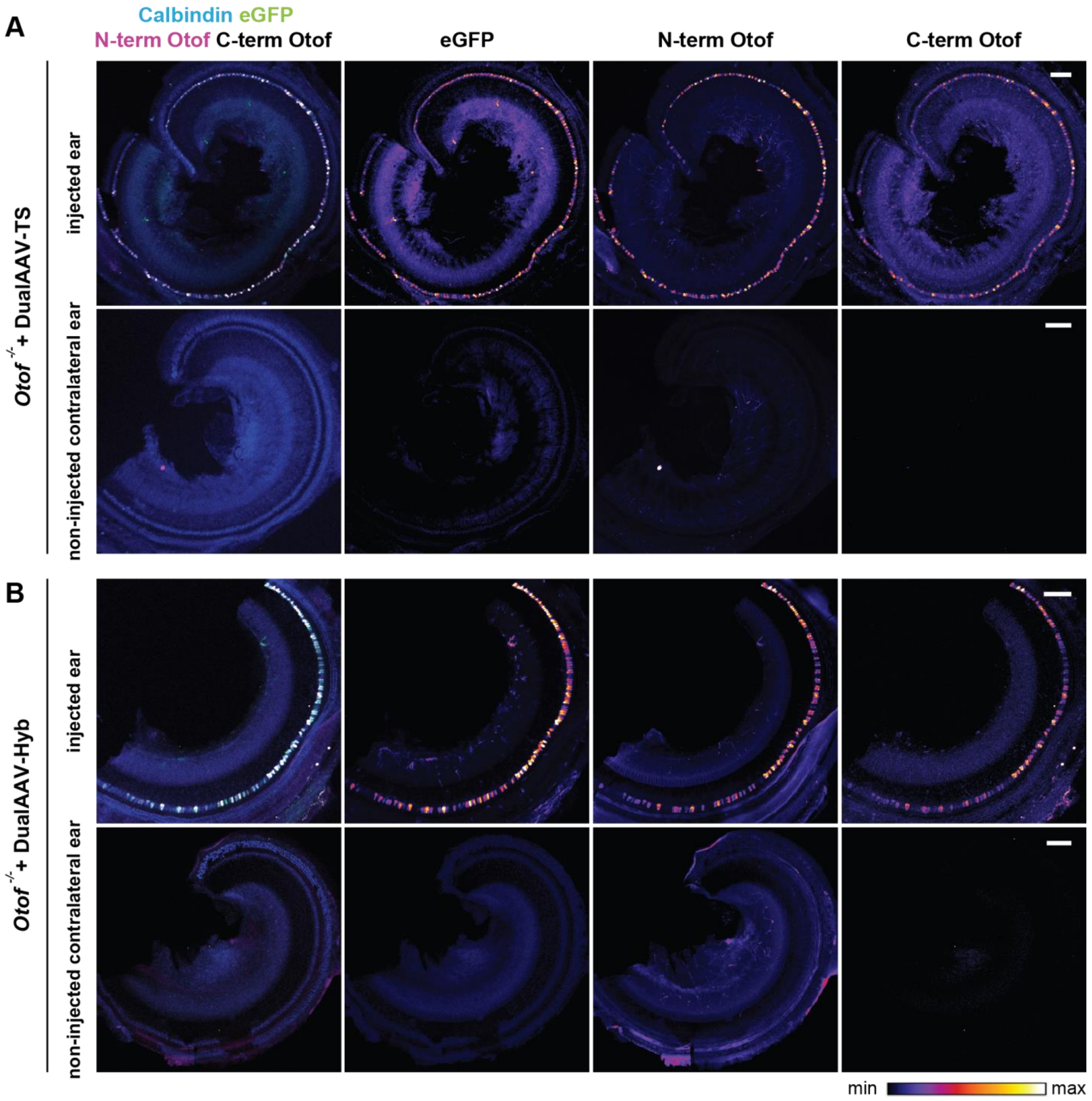

Appendix Figure S1 - Contralateral non-injected *Otof*$^{-/-}$ ears do not show dual-AAV mediated eGFP and otoferlin expression

A,B Low magnification views of organs of Corti from two CD1B6F1 *Otof*$^{-/-}$ mice treated with either otoferlin dual-AAV-TS (**A**; P28) or dual-AAV-Hyb (**B**; P26) half vectors. Both injected (**upper panels**) and contralateral non-injected ears (**lower panels**) of these animals were processed in parallel and acquired and displayed with the same settings. Organs of Corti were co-immunolabeled for calbindin (blue), eGFP (green), and the N-terminal (magenta) and C-terminal (white) part of otoferlin. Individual eGFP, N-terminal otoferlin, and C-terminal otoferlin immunostainings are depicted as color lookup tables with warmer colors representing higher pixel intensities. Maximum intensity projections of optical confocal sections. Scale bars: 100 µm.

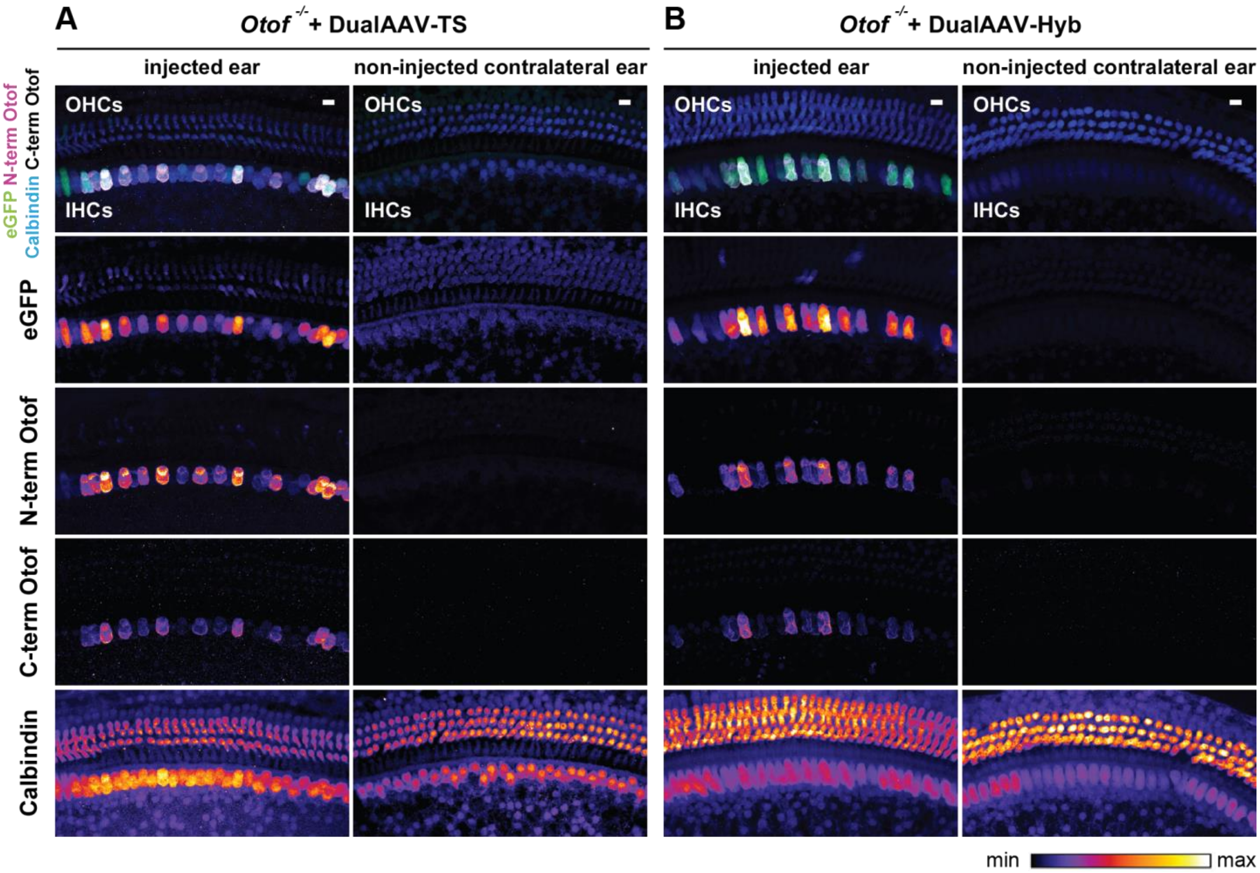

Appendix Figure S2 - OHCs and IHCs from contralateral non-injected *Otof*$^{-/-}$ ears do not show dual-AAV mediated eGFP and otoferlin expression

A,B Higher magnification views of **Appedix Fig S1** showing OHCs and IHCs from two CD1B6F1 *Otof*$^{-/-}$ mice treated with either otoferlin dual-AAV-TS (**A**; P28) or dual-AAV-Hyb (**B**; P26) half vectors. Both injected (**left panels**) and contralateral non-injected ears (**right panels**) of these animals were processed in parallel and acquired and displayed with the same settings. Organs of Corti were co-immunolabeled for eGFP (green) and the N-terminal (magenta) and C-terminal (white) part of otoferlin. OHCs and IHCs were visualized via calbindin immunostaining (blue). Individual eGFP, N-terminal otoferlin, and C-terminal otoferlin immunostainings are depicted as color lookup tables with warmer colors representing higher pixel intensities. Maximum intensity projections of optical confocal sections. IHCs: inner hair cells, OHCs: outer hair cells. Scale bars: Scale bars: 10 µm.

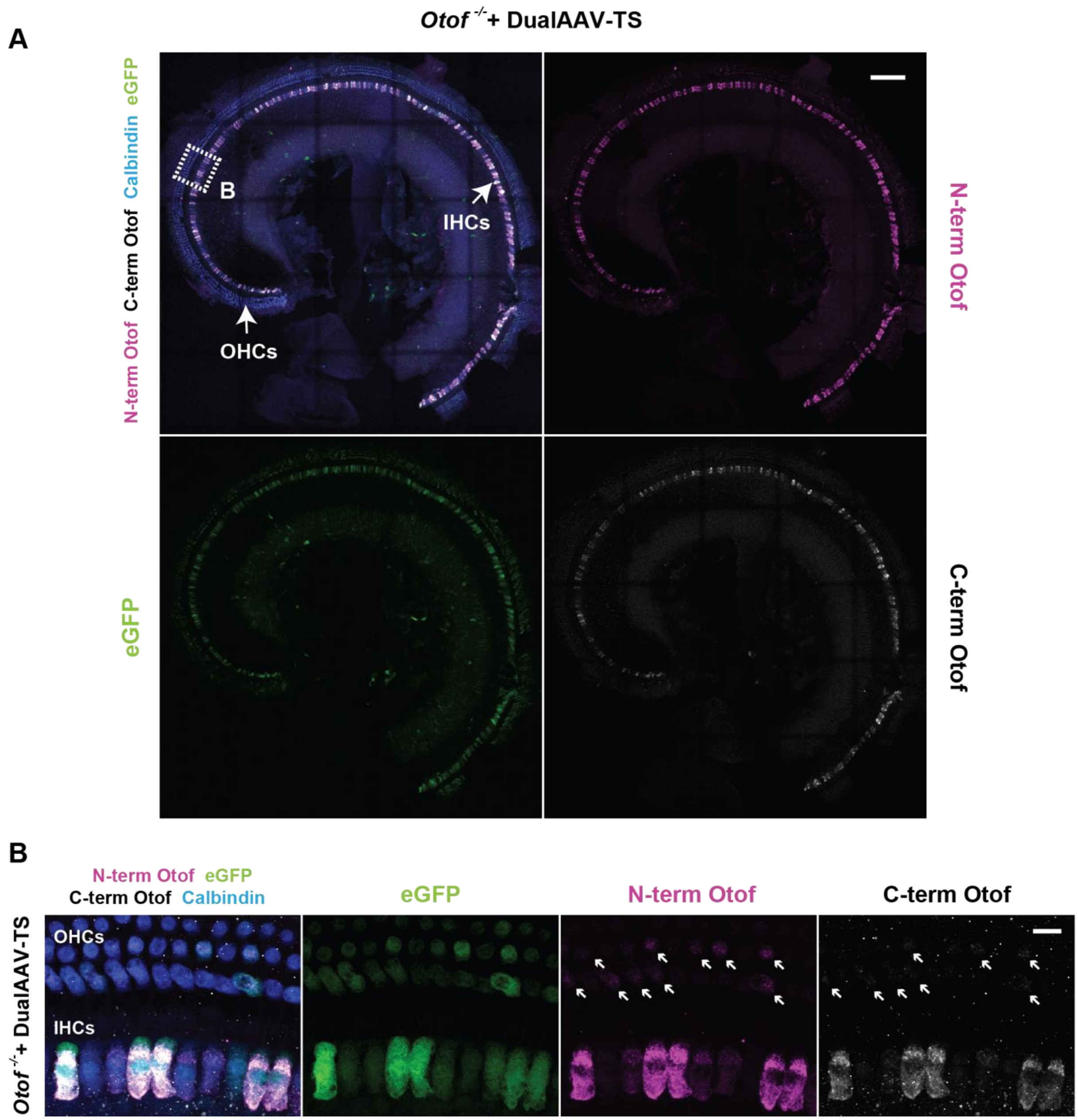

Appendix Figure S3 – OHCs were sparsely transduced with dual-AAV vectors.

A Low magnification views of a CD1 *Otof*$^{-/-}$ organ of Corti (P23) depicted in **Fig 1A** transduced with otoferlin dual-AAV-TS half vectors. The organ of Corti was co-immunolabeled for calbindin (blue), eGFP (green), and the N-terminal (magenta) and C-terminal (white) part of otoferlin.

B Higher magnification views of (**A**) showing OHCs expressing eGFP (green) and otoferlin (magenta, white, arrows) and IHCs transduced with otoferlin dual-AAV-TS half vectors.

Data information: Maximum intensity projections of optical confocal sections. IHCs: inner hair cells, OHCs: outer hair cells. Scale bars: 100µm (A), 10 µm (B).

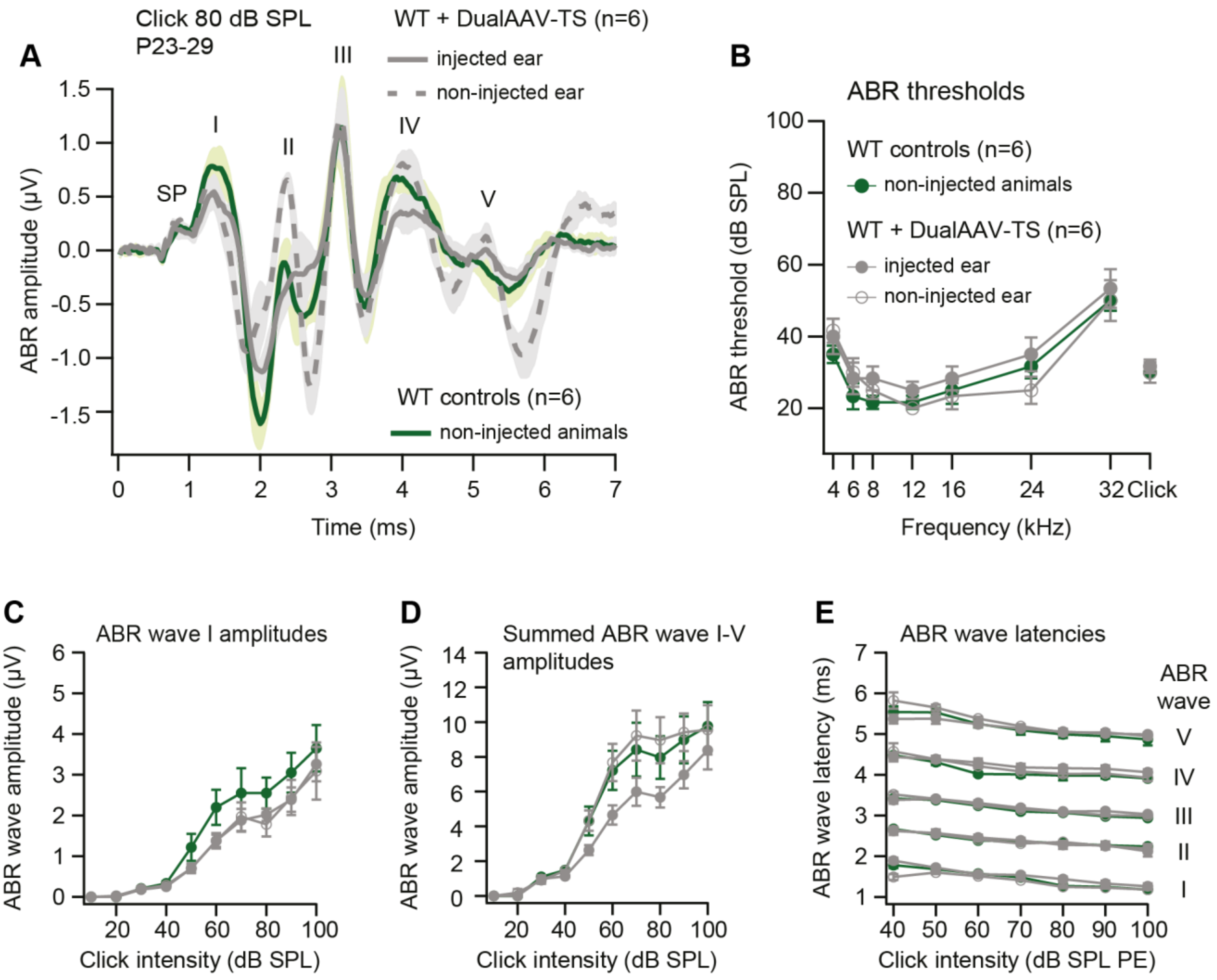

Appendix Figure S4 - Otoferlin dual-AAV-TS injected and non-injected contralateral ears of wild-type mice did not differ in ABR thresholds and wave latencies.

A Average ABR wave responses to 80dB SPL broadband click sound stimuli in otoferlin dual-AAV-TS injected and contralateral non-injected ears of CD1B6F1 wild-type mice (WT+ DualAAV-TS) compared to non-injected control CD1B6F1 wild-type animals (WT controls, P23-29). SP: summating receptor potential, wave I: auditory nerve activity, wave II: cochlear nucleus activity, wave III: superior olivary complex activity, wave IV-V: lateral lemniscus activity in the brainstem.

B ABR click sound (**right**) and tone burst (**left**) thresholds in otoferlin dual-AAV-TS injected and contralateral non-injected ears of wild-type animals compared to non-injected control wild-type mice.

C, D ABR wave I (**C**) and summed ABR wave I-V (**D**) amplitudes at different click sound intensities in otoferlin dual-AAV-TS injected and non-injected contralateral ears of wild-type mice. Non-injected wild-type mice served as controls.

E ABR wave I-V latencies at different click sound intensities measured from otoferlin dual-AAV-TS injected and non-injected contralateral ears of wild-type mice. SPL PE: sound pressure level peak equivalent.

Data information: In (A-E), data are presented as mean ± SEM.

Appendix Table S1. Statistical tests and *P*-values

Fig 1D.

Group compared	Statistical significance	*P* value	Statistical test
DualAAV-TS:<br>N-term Otoferlin<br>vs.<br>C-term Otoferlin	**	0.002	Wilcoxon matched-pair singed rank test
DualAAV-Hyb:<br>N-term Otoferlin<br>vs.<br>C-term Otoferlin	**	0.004	Wilcoxon matched-pair singed rank test
N-term Otoferlin:<br>DualAAV-TS<br>vs.<br>DualAAV-Hyb	*	0.02	Unpaired t-test with Welch's correction
C-term Otoferlin:<br>DualAAV-TS<br>vs.<br>DualAAV-Hyb	*	0.05	Unpaired t-test with Welch's correction

Fig 1E.

Group compared	Statistical significance	*P* value	Statistical test
N-term Otoferlin:			
WTB6 - AAV<br>vs.<br>WTCD1B6F1 + AAV.eGFP	ns	> 0.999999	Kruskal-Wallis test followed by Dunn's multiple comparison test
WTB6 - AAV<br>vs.<br>WTCD1B6F1 + DualAAV-TS	***	$2x10^{-6}$	Kruskal-Wallis test followed by Dunn's multiple comparison test
WTB6 - AAV<br>vs.<br>*Otof*$^{-/-}$ CD1B6F1 + DualAAV-TS	***	0.0008	Kruskal-Wallis test followed by Dunn's multiple comparison test
WTB6 - AAV<br>vs.<br>*Otof*$^{-/-}$ CD1B6F1 + DualAAV-Hyb	***	$< 1x10^{-6}$	Kruskal-Wallis test followed by Dunn's multiple comparison test
WTB6 - AAV<br>vs.<br>*Otof*$^{-/-}$CD1B6F1 - AAV	***	$< 1x10^{-6}$	Kruskal-Wallis test followed by Dunn's multiple comparison test
Otof$^{-/-}$ CD1B6F1 + DualAAV-TS<br>vs.<br>*Otof*$^{-/-}$ CD1B6F1 + DualAAV-Hyb	ns	> 0.999999	Kruskal-Wallis test followed by Dunn's multiple comparison test
Otof$^{-/-}$ CD1B6F1 + DualAAV-TS<br>vs.<br>*Otof*$^{-/-}$ CD1B6F1 - AAV	***	$< 1x10^{-6}$	Kruskal-Wallis test followed by Dunn's multiple comparison test
Otof$^{-/-}$ CD1B6F1 + DualAAV-Hyb<br>vs.<br>*Otof*$^{-/-}$ CD1B6F1 - AAV	***	$< 1x10^{-6}$	Kruskal-Wallis test followed by Dunn's multiple comparison test

C-term Otoferlin:			
WTB6 - AAV<br>vs.<br>WTCD1B6F1 + AAV.eGFP	**	0.004	Kruskal-Wallis test followed by Dunn's multiple comparison test
WTB6 - AAV<br>vs.<br>WTCD1B6F1 + DualAAV-TS	*	0.031	Kruskal-Wallis test followed by Dunn's multiple comparison test
WTB6 - AAV<br>vs.<br>*Otof*$^{-/-}$ CD1B6F1 + DualAAV-TS	***	$1x10^{-5}$	Kruskal-Wallis test followed by Dunn's multiple comparison test
WTB6 - AAV<br>vs.<br>*Otof*$^{-/-}$ CD1B6F1 + DualAAV-Hyb	***	$< 1x10^{-6}$	Kruskal-Wallis test followed by Dunn's multiple comparison test
WTB6 - AAV<br>vs.<br>*Otof*$^{-/-}$ CD1B6F1 - AAV	***	$< 1x10^{-6}$	Kruskal-Wallis test followed by Dunn's multiple comparison test
Otof$^{-/-}$ CD1B6F1 + DualAAV-TS<br>vs.<br>*Otof*$^{-/-}$ CD1B6F1 + DualAAV-Hyb	ns	> 0.999999	Kruskal-Wallis test followed by Dunn's multiple comparison test
Otof$^{-/-}$CD1B6F1 + DualAAV-TS<br>vs.<br>*Otof*$^{-/-}$ CD1B6F1 - AAV	***	$< 1x10^{-6}$	Kruskal-Wallis test followed by Dunn's multiple comparison test
Otof$^{-/-}$CD1B6F1 + DualAAV-Hyb<br>vs.<br>*Otof*$^{-/-}$CD1B6F1 - AAV	***	$< 1x10^{-6}$	Kruskal-Wallis test followed by Dunn's multiple comparison test

Fig 2B.

Group compared	Statistical significance	*P* value	Statistical test
WTB6<br>vs.<br>WTCD1B6F1 +AAV	ns	> 0.999999	Kruskal-Wallis test followed by Dunn's multiple comparison test
WTB6<br>vs.<br>*Otof*$^{-/-}$+ DualAAV-TS	***	$< 1x10^{-6}$	Kruskal-Wallis test followed by Dunn's multiple comparison test
WTB6<br>vs.<br>*Otof*$^{-/-}$ + DualAAV-Hyb	***	$< 1x10^{-6}$	Kruskal-Wallis test followed by Dunn's multiple comparison test
WTB6<br>vs.<br>*Otof*$^{-/-}$ - AAV<br>injected ear	***	$< 1x10^{-6}$	Kruskal-Wallis test followed by Dunn's multiple comparison test
WTB6<br>vs.<br>*Otof*$^{-/-}$ - AAV<br>non-injected ear	***	$< 1x10^{-6}$	Kruskal-Wallis test followed by Dunn's multiple comparison test
WTCD1B6F1 + AAV<br>vs.<br>*Otof*$^{-/-}$ + DualAAV-TS	***	$< 1x10^{-6}$	Kruskal-Wallis test followed by Dunn's multiple comparison test

WTCD1B6F1 + AAV vs. *Otof*$^{-/-}$ + DualAAV-Hyb	***	< $1x10^{-6}$	Kruskal-Wallis test followed by Dunn's multiple comparison test
WTCD1B6F1 + AAV vs. *Otof*$^{-/-}$ - AAV injected ear	***	< $1x10^{-6}$	Kruskal-Wallis test followed by Dunn's multiple comparison test
WTCD1B6F1 + AAV vs. *Otof*$^{-/-}$ - AAV non-injected ear	***	< $1x10^{-6}$	Kruskal-Wallis test followed by Dunn's multiple comparison test
Otof$^{-/-}$ + DualAAV-TS vs. *Otof*$^{-/-}$ + DualAAV-Hyb	ns	> 0.999999	Kruskal-Wallis test followed by Dunn's multiple comparison test
Otof$^{-/-}$ + DualAAV-TS vs. *Otof*$^{-/-}$ - AAV injected ear	ns	> 0.999999	Kruskal-Wallis test followed by Dunn's multiple comparison test
Otof$^{-/-}$ + DualAAV-TS vs. *Otof*$^{-/-}$ - AAV non-injected ear	ns	> 0.999999	Kruskal-Wallis test followed by Dunn's multiple comparison test
Otof$^{-/-}$ + DualAAV-Hyb vs. *Otof*$^{-/-}$ - AAV injected ear	ns	0.32	Kruskal-Wallis test followed by Dunn's multiple comparison test
Otof$^{-/-}$ + DualAAV-Hyb vs. *Otof*$^{-/-}$ - AAV non-injected ear	ns	> 0.999999	Kruskal-Wallis test followed by Dunn's multiple comparison test
Otof$^{-/-}$ - AAV injected ear vs. *Otof*$^{-/-}$ - AAV non-injected ear	ns	> 0.999999	Kruskal-Wallis test followed by Dunn's multiple comparison test

Fig 2D.

Group compared	Statistical significance	*P* value	Statistical test
WT B6 P6 vs. *Otof*$^{-/-}$ B6 P6	**	0.002	Kruskal-Wallis test followed by Dunn's multiple comparison test
WT B6 P6 vs. WT B6 P14	***	< $1x10^{-6}$	Kruskal-Wallis test followed by Dunn's multiple comparison test
WT B6 P6 vs. *Otof*$^{-/-}$ B6 P14	***	< $1x10^{-6}$	Kruskal-Wallis test followed by Dunn's multiple comparison test

Otof$^{-/-}$ B6 P6 vs. WT B6 P14	***	< 1x10^{-6}	Kruskal-Wallis test followed by Dunn's multiple comparison test
Otof$^{-/-}$ B6 P6 vs. *Otof*$^{-/-}$ B6 P14	***	< 1x10^{-6}	Kruskal-Wallis test followed by Dunn's multiple comparison test
WT B6 P14 vs. *Otof*$^{-/-}$ B6 P14	ns	0.37	Kruskal-Wallis test followed by Dunn's multiple comparison test

Fig 2G.

20 ms depolarization			
Group compared	Statistical significance	*P* value	Statistical test
WTB6 vs. WTCD1B6F1 vs. *Otof*$^{-/-}$+ DualAAV-TS	ns	0.3	One-way ANOVA
Otof$^{-/-}$+ DualAAV-TS vs. *Otof*$^{-/-}$	***	<1x10^{-4}	t-test (unpaired, two-tailed)
20-100ms vesicle replenishment rate			
Otof$^{-/-}$ + DualAAV-TS vs. *Otof*$^{-/-}$	***	< 1x10^{-4}	t-test (two-tailed, unpaired)
WTCD1B6F1 vs. *Otof*$^{-/-}$ + DualAAV-TS	***	0.0005	One-way ANOVA followed by Sidak's multiple comparisons test
WTB6 vs. *Otof*$^{-/-}$ + DualAAV-TS	***	<1x10^{-4}	One-way ANOVA followed by Sidak's multiple comparisons test

Fig 3C.

Group compared	Statistical significance	*P* value	Statistical test
WT + AAV.eGFP vs. WT + DualAAV-TS	ns	> 0.999999	Kruskal-Wallis test followed by Dunn's multiple comparison test
WT + AAV.eGFP vs. *Otof*$^{-/-}$ + DualAAV-TS	***	0.00002	Kruskal-Wallis test followed by Dunn's multiple comparison test
WT + AAV.eGFP vs. *Otof*$^{-/-}$ + DualAAV-Hyb	***	0.00013	Kruskal-Wallis test followed by Dunn's multiple comparison test
Otof$^{-/-}$ + DualAAV-TS vs. *Otof*$^{-/-}$ + DualAAV-Hyb	ns	> 0.999999	Kruskal-Wallis test followed by Dunn's multiple comparison test

Fig 3F.

Group	Statistical significance	*P* value + correlation coefficient	Correlation test
50 dB SPL	ns	*P* = 0.24<br>r = 0.54	Spearman correlation test
70 dB SPL	*	*P* = 0.04<br>r = 0.78	Pearson correlation test
90 dB SPL	*	*P* = 0.02<br>r = 0.84	Pearson correlation test

Fig EV5C.

Group	Statistical significance	*P* value + correlation coefficient	Correlation test
Otof$^{-/-}$ + DualAAV-TS	ns	*P* = 0.50<br>r = - 0.41	Spearman correlation test
