# Supplementary material for: A dual‐AAV approach restores fast exocytosis and partially rescues auditory function in deaf otoferlin knock‐out mice
Source: EMBO Mol Med. 2018 Dec 3;11(1):e9396. doi: 10.15252/emmm.201809396 (PMC6328916; doi:10.15252/emmm.201809396)
Supplement: Supplementary file 2 — Expanded View Figures PDF [file EMMM-11-e9396-s002.pdf]

Expanded View Figures

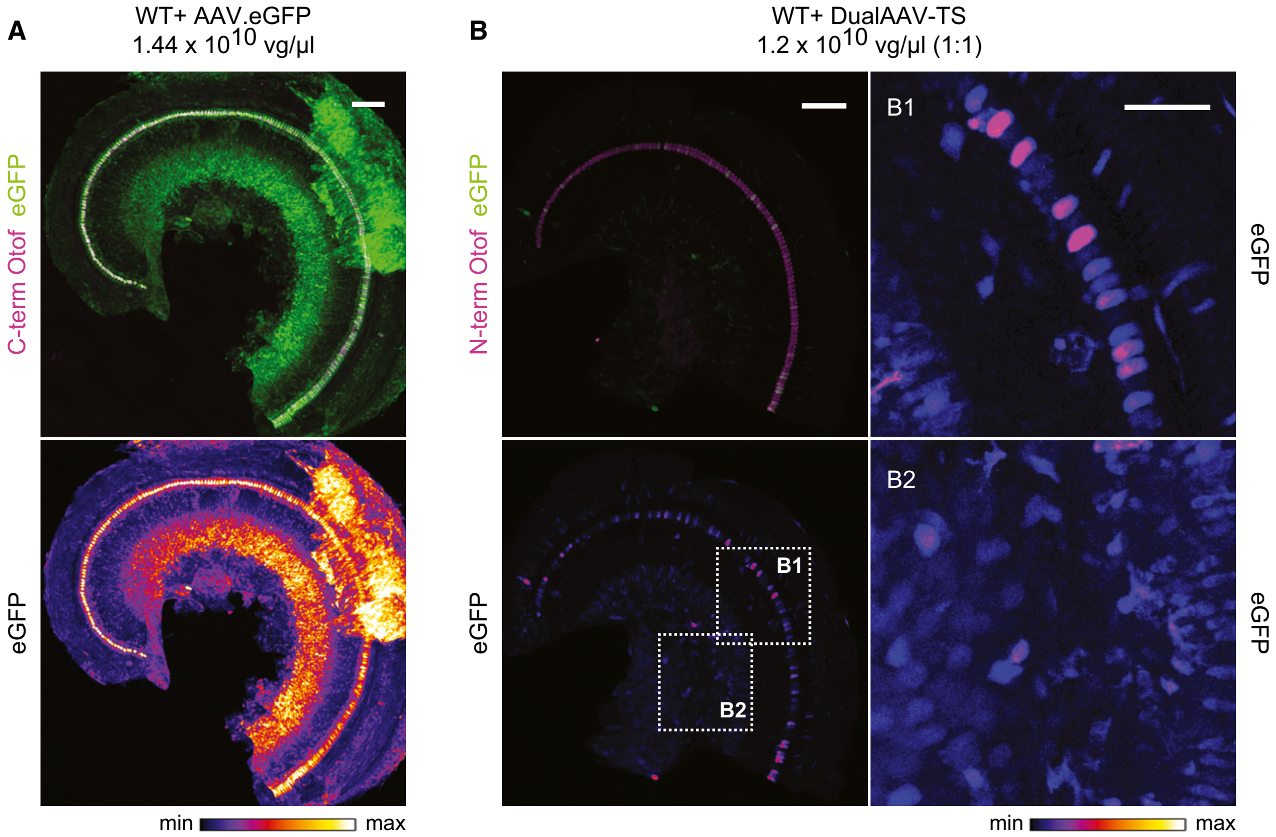

Figure EV1. AAV2/6 transduces various cell types in the inner ear.

A, B Low magnification views for eGFP immunofluorescence in CD1B6F1 wild-type organs of Corti transduced with AAV2/6 vectors, indicating a broad cell type tropism both for a single eGFP-expressing AAV2/6 (A; P23) and eGFP expressed from otoferlin dual-AAV-TS vectors (B; P27). Images were acquired and displayed with the same settings. Organs of Corti were co-immunolabeled for otoferlin (magenta) to visualize IHCs. (B1, B2) High magnification views of (B) displayed with higher intensity showing eGFP immunofluorescence in IHCs and supporting cells (B1) and in spiral ganglion neurons (B2) in dual-AAV-TS-treated wild-type mice. Individual eGFP immunostainings were depicted as color lookup tables with warmer colors representing higher pixel intensities (max). Maximum intensity projections of optical confocal sections. Vg, vector genomes. Scale bars: 100 µm (A, B), 50 µm (B1, B2).

 © 2018 The Authors

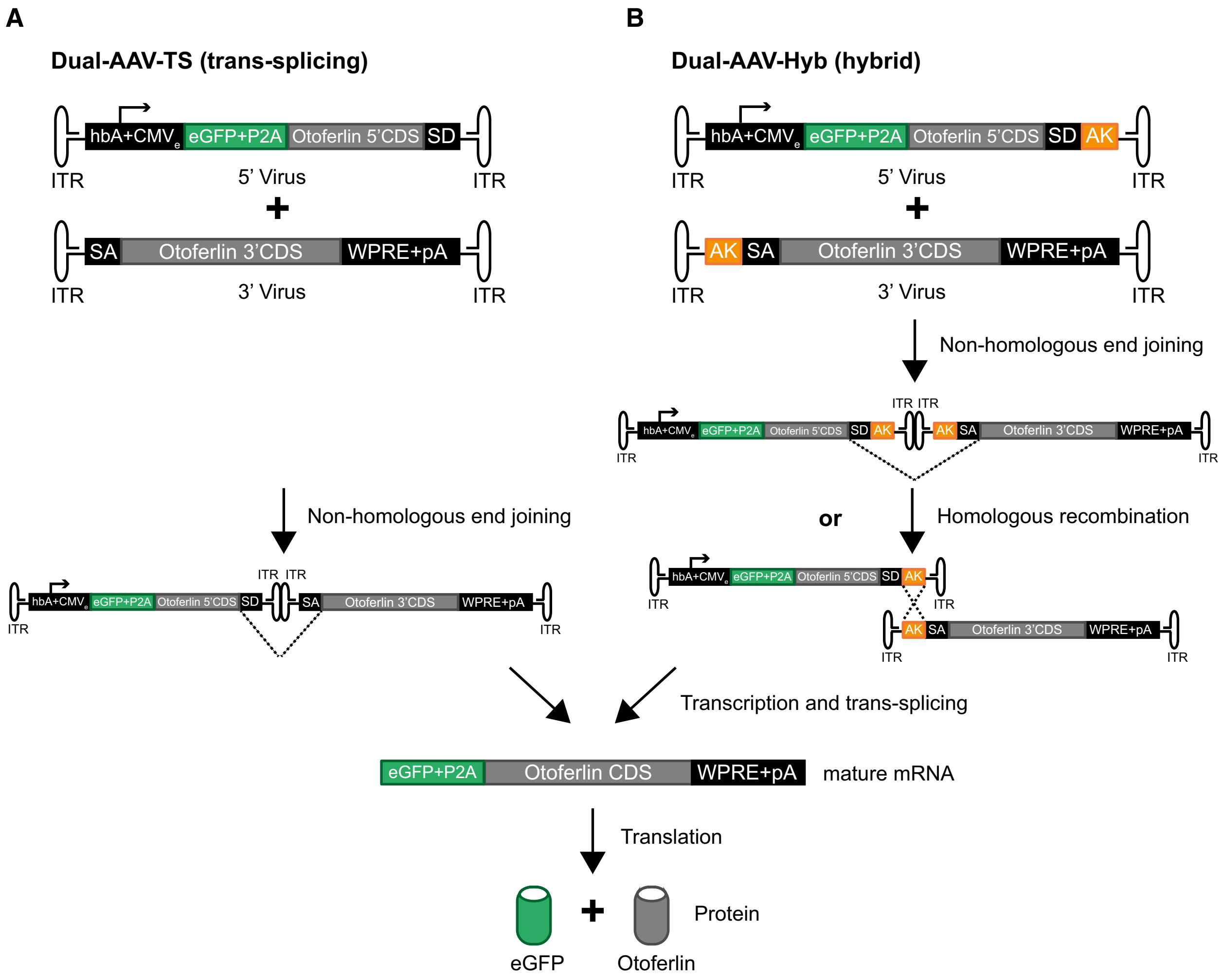

Figure EV2. Dual-AAV vector strategies for full-length otoferlin gene transfer.

A, B Schematic representation of the otoferlin dual-AAV-TS (A) and dual-AAV-Hyb (B) half-vectors used for postnatal cochlear injections. Both otoferlin dual-AAV half-vector systems contain the first half of the otoferlin coding sequence (CDS) in the 5′-AAV and the other half in the 3′-AAV half-vector. The correct reconstitution of the full-length otoferlin mRNA in the dual-AAV-TS strategy is mediated by non-homologous end joining of the inverted terminal repeats (ITRs). In the dual-AAV-Hyb strategy, the reassembly is mediated by non-homologous end joining of the ITRs and/or homologous recombination of the highly recombinogenic AK sequence. Splice donor (SD) and splice acceptor (SA) sites facilitate the excision of the ITRs via trans-splicing. The woodchuck hepatitis virus post-transcriptional regulatory element (WPRE) stabilizes the mRNA. To ensure the production of two separate proteins, a P2A peptide inducing ribosomal skipping is introduced between the eGFP and the otoferlin CDS. hbA: human beta-actin promoter, CMVe: cytomegalovirus enhancer, pA: polyadenylation signal.

© 2018 The Authors

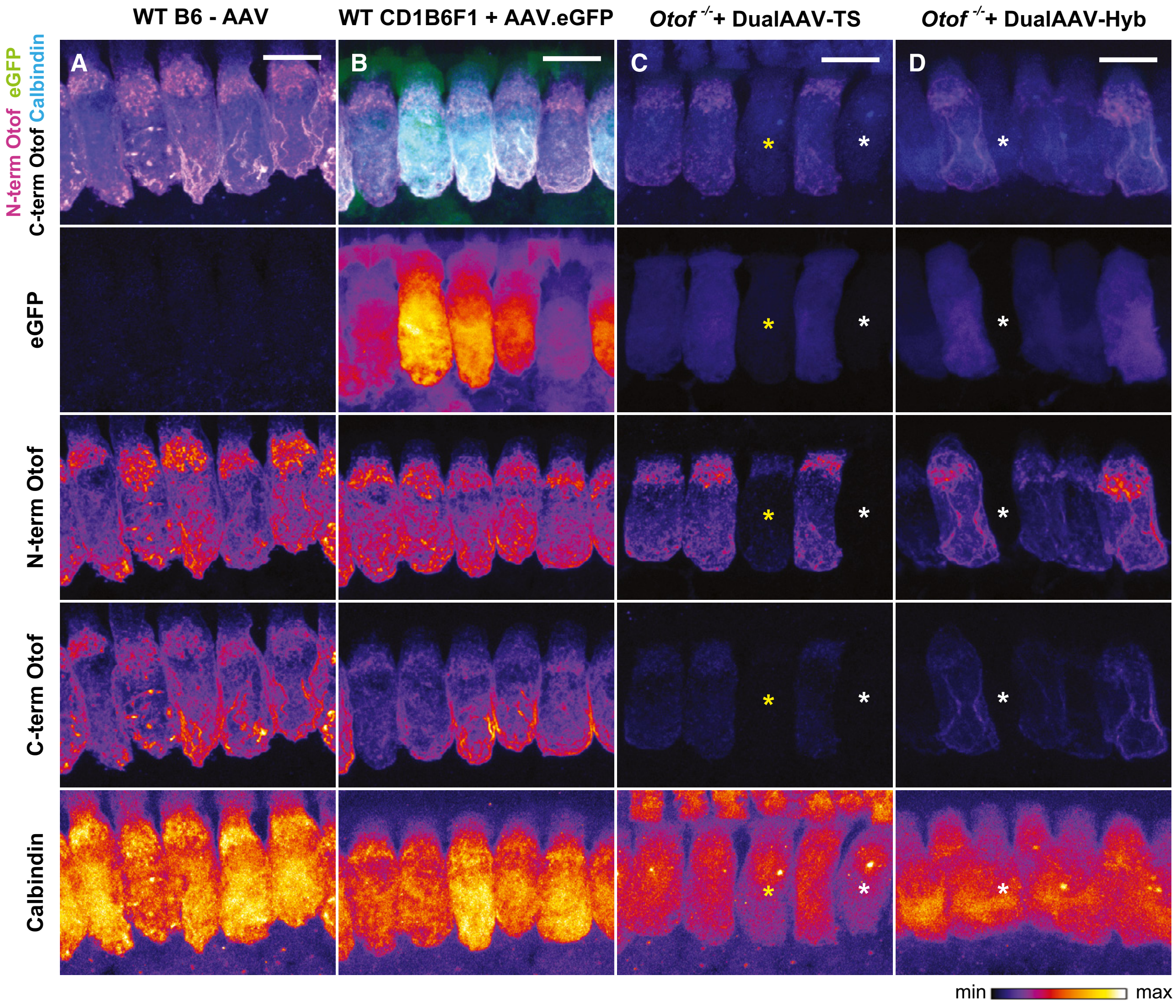

Figure EV3. Cellular localization of eGFP and otoferlin in dual-AAV-TS and dual-AAV-Hyb-transduced *Otof*$^{-/-}$ IHCs compared to wild-type IHCs.

A–D High magnification views of dual-AAV-TS (C; P26) and dual-AAV-Hyb (D; P26)-transduced CD1B6F1-*Otof*$^{-/-}$ IHCs depicted in Fig 1B and C and compared to AAV2/6.eGFP transduced CD1B6F1 wild-type (B; P28) and non-injected B6 wild-type (A; P27) IHCs. Successful virus transduction is monitored via eGFP immunofluorescence. Organs of Corti were immunolabeled against the N-terminal (magenta) and C-terminal (white) part of otoferlin. HCs were immunolabeled with calbindin. Individual eGFP, otoferlin, and calbindin immunostainings are depicted as color lookup tables with warmer colors representing higher pixel intensities. Non-transduced IHCs are labeled with white asterisks, and one transduced IHC displaying only eGFP and N-terminal otoferlin fluorescence, but hardly any C-terminal otoferlin fluorescence with a yellow asterisk. All samples were processed in parallel and acquired and displayed with the same settings. Maximum intensity projections of optical confocal sections. Scale bars: 10 μm.

 © 2018 The Authors

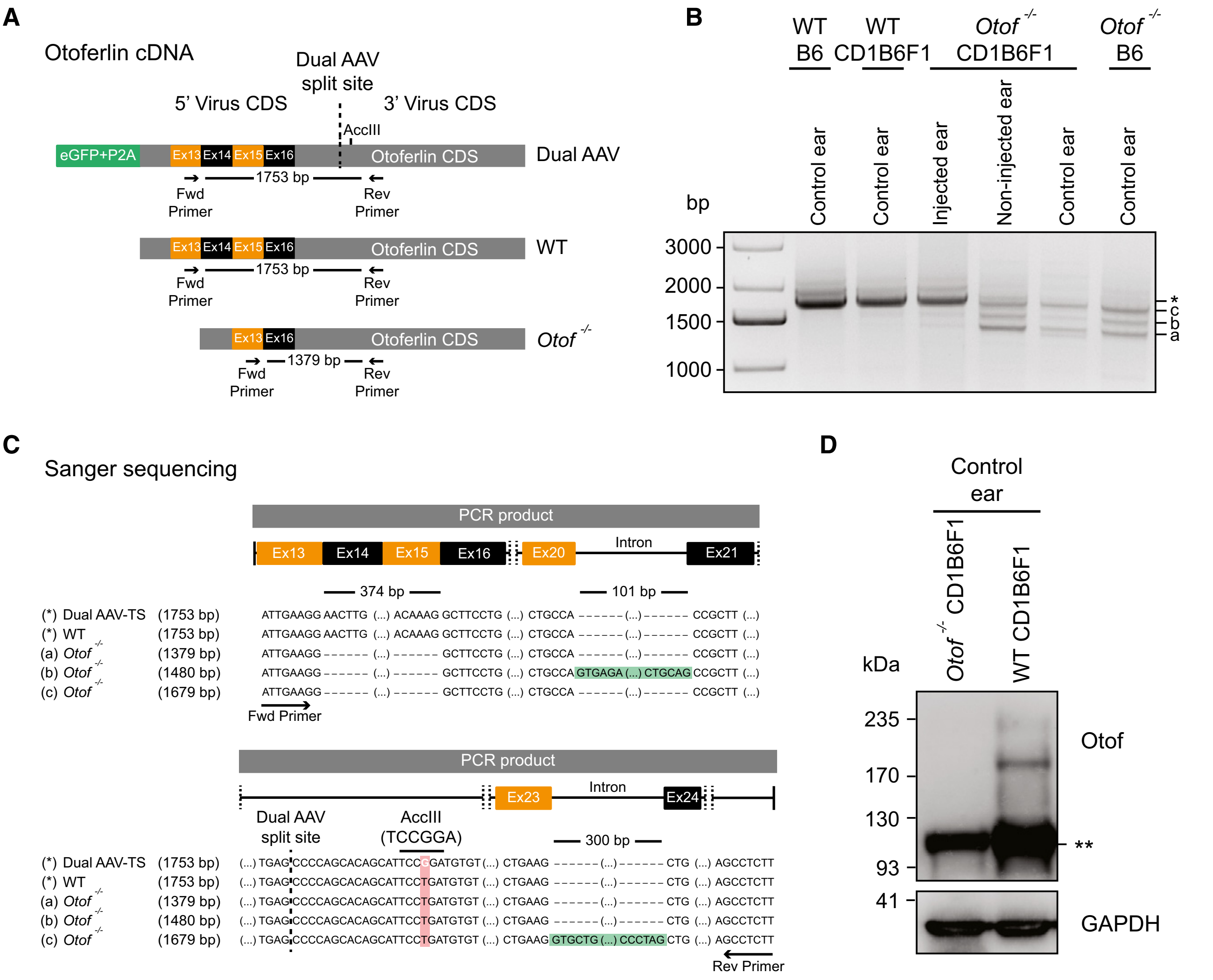

Figure EV4. Otoferlin dual-AAV-TS-transduced *Otof*$^{-/-}$ organs of Corti express full-length otoferlin mRNA.

A Schematic representation of otoferlin cDNA from otoferlin dual-AAV-transduced, wild-type, and *Otof*$^{-/-}$ organs of Corti, displaying binding sites of primers used in PCRs to assess dual-AAV reassembly.

B Otoferlin PCR amplicons from organ of Corti cDNA. A 1,753-bp-long amplicon (*), also present in non-injected wild-type controls (WTB6, WTCD1B6F1), indicates successful reassembly of the split otoferlin expression cassette in otoferlin dual-AAV-TS-transduced CD1B6F1-*Otof*$^{-/-}$ organs of Corti (injected ear). In *Otof*$^{-/-}$ samples, three shorter products were amplified (a, b and c).

C Sanger sequencing confirmed correct dual-AAV split-site assembly (dashed line) as well as the presence of an artificial AccIII restriction site introduced in the dual-AAV-TS otoferlin cDNA, which is absent in the wild-type (WT) and *Otof*$^{-/-}$ cDNA (a–c). Amplicons a-c from *Otof*$^{-/-}$ organs of Corti all lack exons 14–15, while bands "b" (1,480 bp) and "c" (1,679 bp) still contain intron 20–21 (b) or intron 23–24 (c), respectively.

D Western blotting on cell lysates of WT and *Otof*$^{-/-}$ CD1B6F1 organs of Corti. Two bands of ~210–230 kDa, corresponding to full-length otoferlin, were detected in WT but absent in *Otof*$^{-/-}$ ears. (**) refers to an unspecific band detected in both samples. GAPDH was used as loading control.

Data information: CDS: coding sequence, Ex: exon, TS: trans-splicing, Hyb: hybrid, control ear: non-treated ears, non-injected ear: contralateral non-injected *Otof*$^{-/-}$ ears. Source data are available online for this figure.

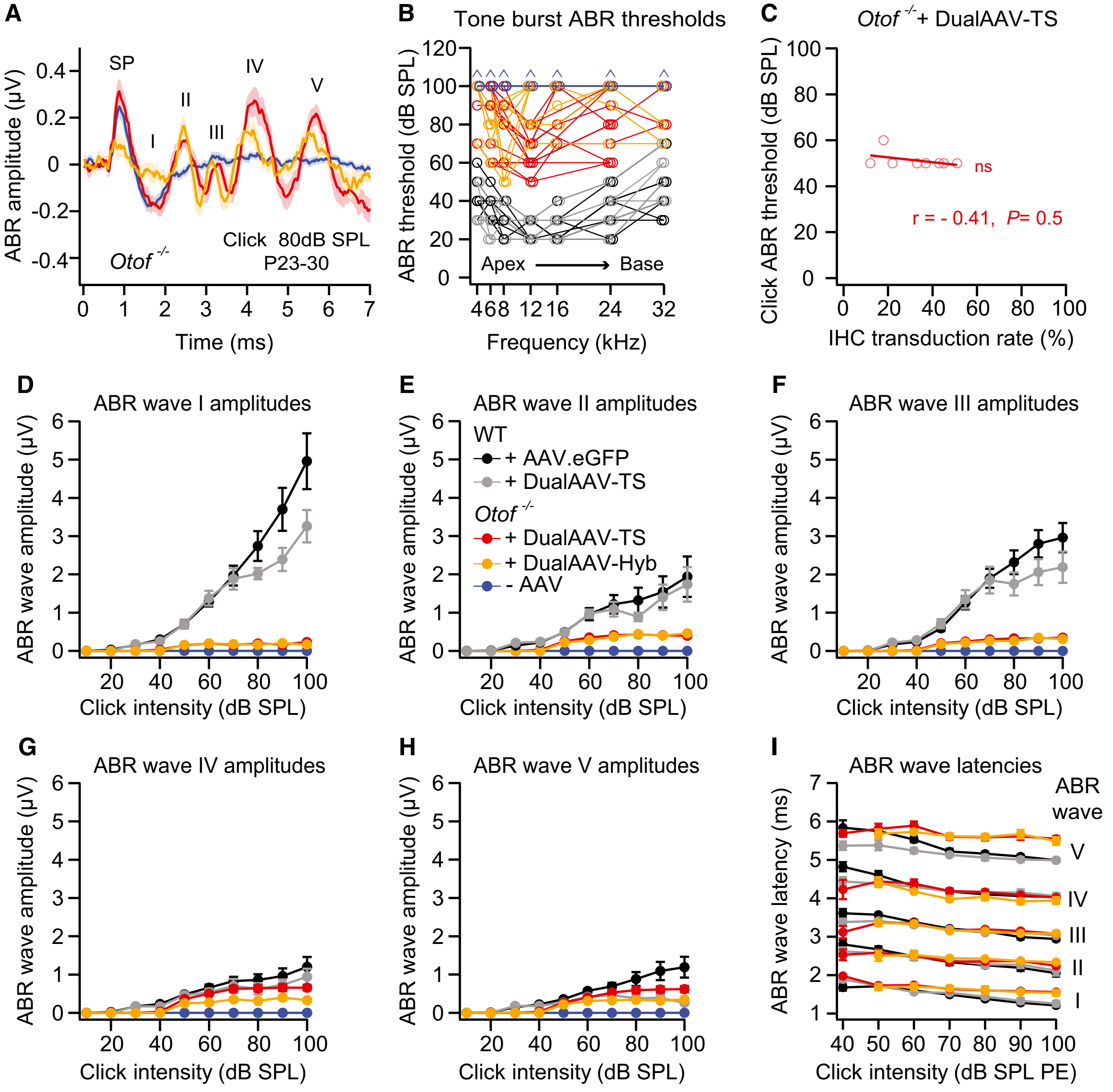

Figure EV5. Otoferlin dual-AAV-treated *Otof*$^{-/-}$ mice show partially restored ABR wave amplitudes.

A Average ABR waves evoked by 80 dB SPL click sound stimuli in otoferlin dual-AAV-injected compared to non-injected deaf CD1B6F1 *Otof*$^{-/-}$ mice. SP: summating potential.

B Tone burst ABR thresholds of individual otoferlin dual-AAV-treated *Otof*$^{-/-}$ mice compared to non-treated *Otof*$^{-/-}$ and wild-type control animals. Animals with thresholds exceeding the maximum loudspeaker output of 90 dB SPL were set to 100 dB SPL (arrows). Apical and basal cochlear turns are indicated as Apex and Base, respectively.

C ABR click sound thresholds of individual dual-AAV-TS injected CD1B6F1-*Otof*$^{-/-}$ animals (n = 8 mice; from Fig 3C) plotted against their full-length otoferlin IHC transduction rates (from Fig 1D, C-term otoferlin). r: correlation coefficient.

D–H ABR wave I (D), wave II (E), wave III (F), wave IV (G), and wave V (H) amplitudes, calculated as the difference between the local maximum and the subsequent local minimum, at different click sound intensities in otoferlin dual-AAV-injected and non-injected CD1B6F1-*Otof*$^{-/-}$ mice compared to AAV2/6.eGFP and dual-AAV-TS injected control CD1B6F1 wild-type (WT) animals. SPL: sound pressure level.

I ABR wave I-V latencies at different click sound intensities in otoferlin dual-AAV-injected *Otof*$^{-/-}$ and injected CD1B6F1 wild-type control mice. PE: peak equivalent.

Data information: In (A–I), age of analyzed animals: P23–30. In (A, D–I), number of analyzed mice: CD1B6F1 wild-type animals (+AAV.eGFP: n = 12 mice, dualAAV-TS: n = 6 mice) and CD1B6F1-*Otof*$^{-/-}$ animals (dualAAV-TS: n = 17 mice, dualAAV-Hyb: n = 8 mice, -AAV: n = 38 mice). In (B), number of analyzed mice is the same as for (D–I) except: CD1B6F1-*Otof*$^{-/-}$ animals (dualAAV-TS: n = 16 mice). In (B, C), individual animals are depicted with open symbols. In (A, D–I), data are represented as mean ± s.e.m. In (C), $0.5 > r > -0.5$ no correlation (Spearman correlation test).

 © 2018 The Authors
