# Supplementary material for: A dual‐AAV approach restores fast exocytosis and partially rescues auditory function in deaf otoferlin knock‐out mice
Source: EMBO Mol Med. 2018 Dec 3;11(1):e9396. doi: 10.15252/emmm.201809396 (PMC6328916; doi:10.15252/emmm.201809396)
Supplement: Supplementary file 3 — Source Data for Expanded View [file EMMM-11-e9396-s003.zip › Fig_EV4_source_data/EMM-2018-09396-V2-FigEV4C_PCR_ampliconAlignment.pdf]

Alignment

In this file we show an alignment of the sequencing results with the full-length otoferlin sequences of both dual AAV CDS and wild-type variant 4. See below a detailed description of all the sequences.

Full-length sequences:

- **DualAAV mOtof CDS** – full-length mouse otoferlin sequence used to clone the AAV constructs
- **WT mOtof var4 CDS** – full-length otoferlin variant 4 (NM_001313767.1), present in our wild-type mice

Sequencing results:

- **(*) DualAAV-TS (1753 bp)** - representative sequencing result of band * (1753 bp) for the injected ear of *Otof-/-* mice in figure EV4, B
- **(*) WT (1753 bp)** - representative sequencing result of band * (1753 bp) for the control ear of wild-type mice in figure EV4, B
- **(c) Otof-/- (1679 bp)** – representative sequencing result of band c (1679 bp) in figure EV4, B
- **(b) Otof-/- (1480 bp)** – representative sequencing result of band b (1480 bp) in figure EV4, B
- **(a) Otof-/- (1379 bp)** – representative sequencing result of band a (1379 bp) in figure EV4, B

The 101 bp additional sequence in b (Seq 6 in alignment) is the intron between Exon 20 and 21.

The 300 bp additional sequence in c (Seq 5 in alignment) is the intron between Exon 23 and 24.

bp DNA linear bp DNA circular bp DNA circular bp DNA circular

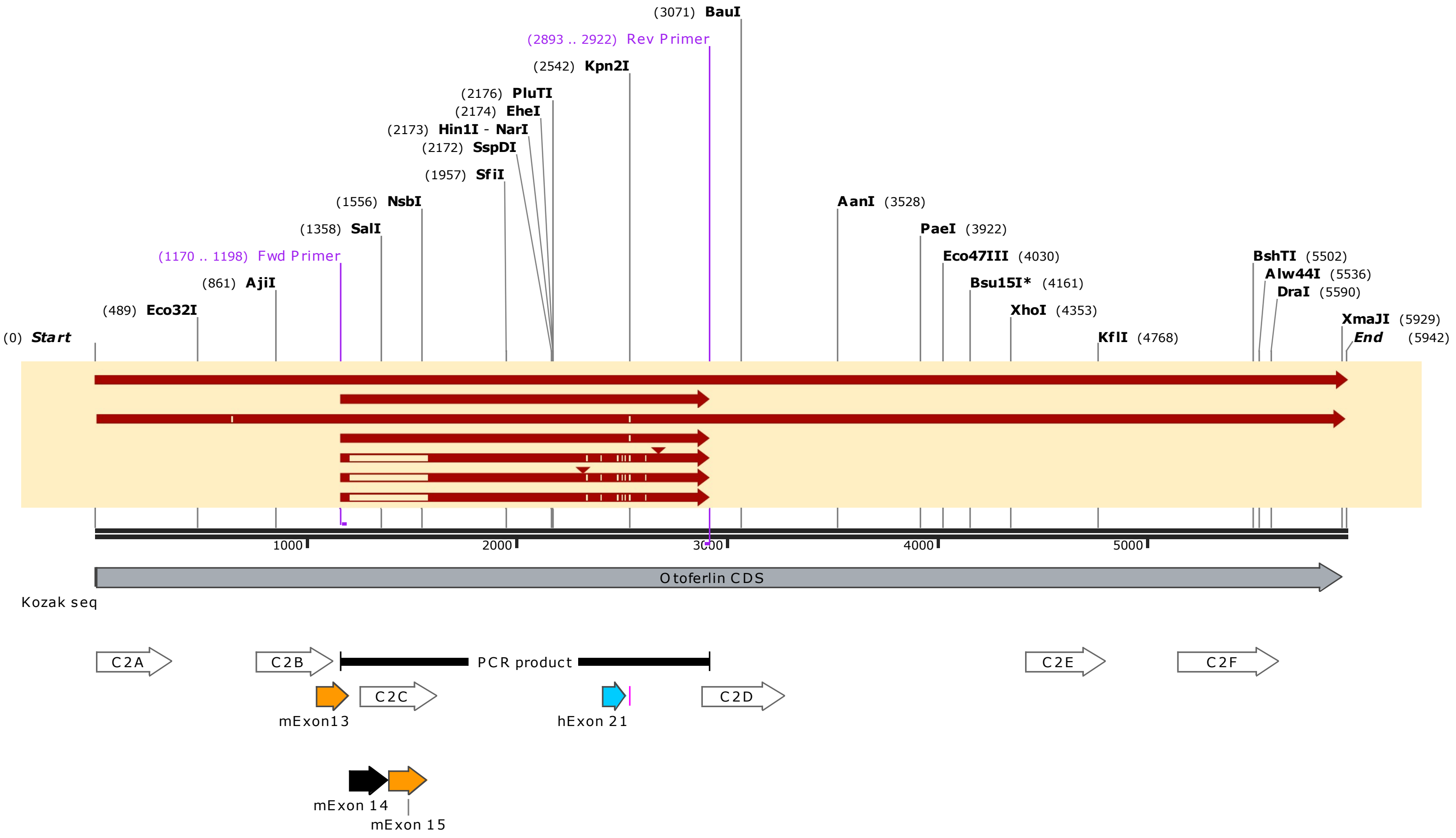

Alignment
5942 bp

10 20 30 40 50 60 70 80 90 100 110

Start (0)

5′ cgccaccatggccctgattgttcacctcaagactgtctcagagctccgaggcaaaggtgaccggattgccaaagtcactttccgagggcagtctttctactcccgggtcc

3′ gcggtggtaccgggactaacaagtggagttctgacagagtctcgaggctccgtttccactggcctaacggtttcagtgaaaggctcccgtcagaaagatgagggcccagg

Kozak seq

Otoferlin CDS

C2A

cgccaccatggccctgattgttcacctcaagactgtctcagagctccgaggcaaaggtgaccggattgccaaagtcactttccgagggcagtctttctactcccgggtcc

1 cgccaccatggccctgattgttcacctcaagactgtctcagagctccgaggcaaaggtgaccggattgccaaagtcactttccgagggcagtctttctactcccgggtcc

3 ATGGCCCTGATTGTTCACCTCAAGACTGTCTCAGAGCTCCGAGGCAAAGGTGACCGGATTGCCAAAGTCACTTTCCGAGGGCAGTCTTTCTACTCCCGGGTCC

120 130 140 150 160 170 180 190 200 210 220

tggagaactgcgagggtgtggctgactttgatgagacgttccggtggccagtggccagcagcatcgaccggaatgaagtgttggagattcagattttcaactacagcaaa

acctcttgacgctcccacaccgactgaaactactctgcaaggccaccggtcaccggtcgtcgtagctggccttacttcacaacctctaagtctaaaagttgatgtcgttt

Otoferlin CDS

C2A

tggagaactgcgagggtgtggctgactttgatgagacgttccggtggccagtggccagcagcatcgaccggaatgaagtgttggagattcagattttcaactacagcaaa

1 tggagaactgcgagggtgtggctgactttgatgagacgttccggtggccagtggccagcagcatcgaccggaatgaagtgttggagattcagattttcaactacagcaaa

3 TGGAGAACTGCGAGGGTGTGGCTGACTTTGATGAGACGTTCCGGTGGCCAGTGGCCAGCAGCATCGACCGGAATGAAGTGTTGGAGATTCAGATTTTCAACTACAGCAAA

230 240 250 260 270 280 290 300 310 320 330

gtcttcagcaacaagctgatagggaccttctgcatggtgctgcagaaagtggtggaggagaatcgggtagaggtgaccgacacgctgatggatgacagcaatgctatcat

cagaagtcgttgttcgactatccctggaagacgtaccacgacgtctttcaccacctcctcttagcccatctccactggctgtgcgactacctactgtcgttacgatagta

Otoferlin CDS

C2A

gtcttcagcaacaagctgatagggaccttctgcatggtgctgcagaaagtggtggaggagaatcgggtagaggtgaccgacacgctgatggatgacagcaatgctatcat

1 gtcttcagcaacaagctgatagggaccttctgcatggtgctgcagaaagtggtggaggagaatcgggtagaggtgaccgacacgctgatggatgacagcaatgctatcat

3 GTCTTCAGCAACAAGCTGATAGGGACCTTCTGCATGGTGCTGCAGAAAGTGGTGGAGGAGAATCGGGTAGAGGTGACCGACACGCTGATGGATGACAGCAATGCTATCAT

340 350 360 370 380 390 400 410 420 430 440

caagaccagcctgagcatggaggtccggtatcaggccacagatggcactgtgggcccctgggatgatggagacttcctgggagatgaatccctccaggaggagaaggaca

gttctggtcggactcgtacctccaggccatagtccggtgtctaccgtgacacccggggaccctactacctctgaaggaccctctacttagggaggtcctcctcttcctgt

Otoferlin CDS

C2A

caagaccagcctgagcatggaggtccggtatcaggccacagatggcactgtgggcccctgggatgatggagacttcctgggagatgaatccctccaggaggagaaggaca

1 caagaccagcctgagcatggaggtccggtatcaggccacagatggcactgtgggcccctgggatgatggagacttcctgggagatgaatccctccaggaggagaaggaca

3 CAAGACCAGCCTGAGCATGGAGGTCCGGTATCAGGCCACAGATGGCACTGTGGGCCCCTGGGATGATGGAGACTTCCTGGGAGATGAATCCCTCCAGGAGGAGAAGGACA

450 460 470 480 490 500 510 520 530 540 550

Eco32I

gccaggagacagatgggctgctacctggttcccgacccagcacccggatatctggcgagaagagctttcgcagagcgggaaggagtgtgttctcggccatgaaactcggc

cggtcctctgtctacccgacgatggaccaagggctgggtcgtgggcctatagaccgctcttctcgaaagcgtctcgcccttcctcacacaagagccggtactttgagccg

Otoferlin CDS

gccaggagacagatgggctgctacctggttcccgacccagcacccggatatctggcgagaagagctttcgcagagcgggaaggagtgtgttctcggccatgaaactcggc

1 gccaggagacagatgggctgctacctggttcccgacccagcacccggatatctggcgagaagagctttcgcagagcgggaaggagtgtgttctcggccatgaaactcggc

3 GCCAGGAGACAGATGGGCTGCTACCTGGTTCCCGACCCAGCACCCGGATATCTGGCGAGAAGAGCTTTCGCAGAGCGGGAAGGAGTGTGTTCTCGGCCATGAAACTCGGC

560 570 580 590 600 610 620 630 640 650 660

aaaactcggtcccacaaagaggagccccaaagacaagatgagccagcagtgctggagatggaggacctggaccacctagccattcagctgggggatgggctggaccctga

ttttgagccagggtgtttctcctcggggtttctgttctactcggtcgtcacgacctctacctcctggacctggtggatcggtaagtcgacccccctacccgacctgggact

Otoferlin CDS

aaaactcggtcccacaaagaggagccccaaagacaagatgagccagcagtgctggagatggaggacctggaccacctagccattcagctgggggatgggctggaccctga

1 aaaactcggtcccacaaagaggagccccaaagacaagatgagccagcagtgctggagatggaggacctggaccacctagccattcagctgggggatgggctggaccctga

3 AAAACTCGGTCCCACAAAGAGGAGCCCCAAAGACAAGATGAGCCAGCAGTGCTGGAGATGGAGGACCTGGACCACCTAGCCATTCAGCTGGGGGATGGGCTGGATCCTGA

670 680 690 700 710 720 730 740 750 760 770

```
ctccgtgtctctagcctcggtcaccgctctcaccagcaatgtctccaacaaacggtctaagccagatattaagatggagcccagtgctggaaggcccatggattaccagg
gaggcacagagatcggagccagtggcgagagtggtcgttacagaggttgtttgccagattcggtctataattctacctcgggtcacgaccttccgggtacctaatggtcc
```

Otoferlin CDS
C2B

```
  ctccgtgtctctagcctcggtcaccgctctcaccagcaatgtctccaacaaacggtctaagccagatattaagatggagcccagtgctggaaggcccatggattaccagg
1 ctccgtgtctctagcctcggtcaccgctctcaccagcaatgtctccaacaaacggtctaagccagatattaagatggagcccagtgctggaaggcccatggattaccagg
3 CTCCGTGTCTCTAGCCTCGGTCACCGCTCTCACCAGCAATGTCTCCAACAAACGGTCTAAGCCAGATATTAAGATGGAGCCCAGTGCTGGAAGGCCCATGGATTACCAGG
```

780 790 800 810 820 830 840 850 860 870 880

AjiI

```
tcagcatcacagtgattgaggctcggcagctggtgggcttgaacatggaccctgtggtgtgtgtggaggtgggtgatgacaagaaatacacgtcaatgaaggagtccaca
agtcgtagtgtcactaactccgagccgtcgaccacccgaacttgtacctgggacaccacacacacctccacccactactgttctttatgtgcagttacttcctcaggtgt
```

Otoferlin CDS
C2B

```
  tcagcatcacagtgattgaggctcggcagctggtgggcttgaacatggaccctgtggtgtgtgtggaggtgggtgatgacaagaaatacacgtcaatgaaggagtccaca
1 tcagcatcacagtgattgaggctcggcagctggtgggcttgaacatggaccctgtggtgtgtgtggaggtgggtgatgacaagaaatacacgtcaatgaaggagtccaca
3 TCAGCATCACAGTGATTGAGGCTCGGCAGCTGGTGGGCTTGAACATGGACCCTGTGGTGTGTGTGGAGGTGGGTGATGACAAGAAATACACGTCAATGAAGGAGTCCACA
```

890 900 910 920 930 940 950 960 970 980 990

```
aactgcccttactacaacgagtactttgtcttcgacttccatgtctctcctgatgtcatgtttgacaagatcatcaagatctcggttatccattctaagaacctgcttcg
ttgacgggaatgatgttgctcatgaaacagaagctgaaggtacagagaggactacagtacaaactgttctagtagttctagagccaataggtaagattcttggacgaagc
```

Otoferlin CDS
C2B

```
  aactgcccttactacaacgagtactttgtcttcgacttccatgtctctcctgatgtcatgtttgacaagatcatcaagatctcggttatccattctaagaacctgcttcg
1 aactgcccttactacaacgagtactttgtcttcgacttccatgtctctcctgatgtcatgtttgacaagatcatcaagatctcggttatccattctaagaacctgcttcg
3 AACTGCCCTTACTACAACGAGTACTTTGTCTTCGACTTCCATGTCTCTCCTGATGTCATGTTTGACAAGATCATCAAGATCTCGGTTATCCATTCTAAGAACCTGCTTCG
```

1000 1010 1020 1030 1040 1050 1060 1070 1080 1090 1100

```
gagcggcaccctggtgggttccttcaaaatggatgtggggactgtgtattcccagcctgaacaccagttccatcacaaatgggccatcctgtcagaccccgatgacatct
ctcgccgtgggaccacccaaggaagttttacctacacccctgacacataagggtcggacttgtggtcaaggtagtgtttacccggtaggacagtctggggctactgtaga
```

Otoferlin CDS
C2B
mExon13

```
  gagcggcaccctggtgggttccttcaaaatggatgtggggactgtgtattcccagcctgaacaccagttccatcacaaatgggccatcctgtcagaccccgatgacatct
1 gagcggcaccctggtgggttccttcaaaatggatgtggggactgtgtattcccagcctgaacaccagttccatcacaaatgggccatcctgtcagaccccgatgacatct
3 GAGCGGCACCCTGGTGGGTTCCTTCAAAATGGATGTGGGGACTGTGTATTCCCAGCCTGAACACCAGTTCCATCACAAATGGGCCATCCTGTCAGACCCCGATGACATCT
```

1110 1120 1130 1140 1150 1160 1170 1180 1190 1200 1210

Fwd Primer
cccacaaggccaacgagacggatgaggac

```
ctgctgggttgaagggttatgtaaagtgtgatgtcgctgtggtgggcaagggagacaacatcaagacaccccacaaggccaacgagacggatgaggacgacattgaaggg
gacgacccaacttcccaatacatttcacactacagcgacaccacccgttccctctgttgtagttctgtggggtgttccggttgctctgcctactcctgctgtaacttccc
```

Otoferlin CDS
C2B
PCR product
mExon13
mExon 14

```
  ctgctgggttgaagggttatgtaaagtgtgatgtcgctgtggtgggcaagggagacaacatcaagacaccccacaaggccaacgagacggatgaggacgacattgaaggg
1 ctgctgggttgaagggttatgtaaagtgtgatgtcgctgtggtgggcaagggagacaacatcaagacaccccacaaggccaacgagacggatgaggacgacattgaaggg
2                                                                       cccacaaggccaacgagacggatgaggacgacattgaaggg
3 CTGCTGGGTTGAAGGGTTATGTAAAGTGTGATGTCGCTGTGGTGGGCAAGGGAGACAACATCAAGACACCCCACAAGGCCAACGAGACGGATGAGGACGACATTGAAGGG
4                                                                       cccacaaggccaacgagacggatgaggacgacattgaaggg
5                                                                       cccacaaggccaacgagacggatgaggacgacattgaagg-
6                                                                       cccacaaggccaacgagacggatgaggacgacattgaagg-
7                                                                       cccacaaggccaacgagacggatgaggacgacattgaagg-
```

```
1220 1230 1240 1250 1260 1270 1280 1290 1300 1310 1320

aacttgctgctccccgagggcgtgccccccgaacggcagtgggcacggttctatgtgaaaatttaccgagcagagggactgccccggatgaacacaagcctcatggccaa
ttgaacgacgaggggctcccgcacggggggcttgccgtcacccgtgccaagatacacttttaaatggctcgtctccctgacggggcctacttgtgttcggagtaccggtt
Otoferlin CDS
PCR product
C2C
mExon 14

  aacttgctgctccccgagggcgtgccccccgaacggcagtgggcacggttctatgtgaaaatttaccgagcagagggactgccccggatgaacacaagcctcatggccaa
1 aacttgctgctccccgagggcgtgccccccgaacggcagtgggcacggttctatgtgaaaatttaccgagcagagggactgccccggatgaacacaagcctcatggccaa
2 aacttgctgctccccgagggcgtgccccccgaacggcagtgggcacggttctatgtgaaaatttaccgagcagagggactgccccggatgaacacaagcctcatggccaa
3 AACTTGCTGCTCCCCGAGGGCGTGCCCCCCGAACGGCAGTGGGCACGGTTCTATGTGAAAATTTACCGAGCAGAGGGACTGCCCCGGATGAACACAAGCCTCATGGCCAA
4 aacttgctgctccccgagggcgtgccccccgaacggcagtgggcacggttctatgtgaaaatttaccgagcagagggactgccccggatgaacacaagcctcatggccaa
5 --------------------------------------------------------------------------------------------------------------
6 --------------------------------------------------------------------------------------------------------------
7 --------------------------------------------------------------------------------------------------------------
```

```
1330 1340 1350 1360 1370 1380 1390 1400 1410 1420 1430

SalI
cgtgaagaaggcgttcatcggtgagaacaaggacctcgtcgacccctatgtgcaagtcttctttgctggacaaaagggcaaaacatcagtgcagaagagcagctatgagc
gcacttcttccgcaagtagccactcttgttcctggagcagctggggatacacgttcagaagaaacgacctgttttcccgttttgtagtcacgtcttctcgtcgatactcg
Otoferlin CDS
PCR product
C2C
mExon 14    mExon 15

  cgtgaagaaggcgttcatcggtgagaacaaggacctcgtcgacccctatgtgcaagtcttctttgctggacaaaagggcaaaacatcagtgcagaagagcagctatgagc
1 cgtgaagaaggcgttcatcggtgagaacaaggacctcgtcgacccctatgtgcaagtcttctttgctggacaaaagggcaaaacatcagtgcagaagagcagctatgagc
2 cgtgaagaaggcgttcatcggtgagaacaaggacctcgtcgacccctatgtgcaagtcttctttgctggacaaaagggcaaaacatcagtgcagaagagcagctatgagc
3 CGTGAAGAAGGCGTTCATCGGTGAGAACAAGGACCTCGTCGACCCCTATGTGCAAGTCTTCTTTGCTGGACAAAAGGGCAAAACATCAGTGCAGAAGAGCAGCTATGAGC
4 cgtgaagaaggcgttcatcggtgagaacaaggacctcgtcgacccctatgtgcaagtcttctttgctggacaaaagggcaaaacatcagtgcagaagagcagctatgagc
5 --------------------------------------------------------------------------------------------------------------
6 --------------------------------------------------------------------------------------------------------------
7 --------------------------------------------------------------------------------------------------------------
```

```
1440 1450 1460 1470 1480 1490 1500 1510 1520 1530 1540

cgctatggaatgagcaggtcgtcttcacagacttgttcccccccactctgcaaacgcatgaaggtgcagatccgggactctgacaaggtcaatgatgtggccatcggcacc
gcgataccttactcgtccagcagaagtgtctgaacaagggggggtgagacgtttgcgtacttccacgtctaggccctgagactgttccagttactacaccggtagccgtgg
Otoferlin CDS
PCR product
C2C
mExon 15

  cgctatggaatgagcaggtcgtcttcacagacttgttcccccccactctgcaaacgcatgaaggtgcagatccgggactctgacaaggtcaatgatgtggccatcggcacc
1 cgctatggaatgagcaggtcgtcttcacagacttgttcccccccactctgcaaacgcatgaaggtgcagatccgggactctgacaaggtcaatgatgtggccatcggcacc
2 cgctatggaatgagcaggtcgtcttcacagacttgttcccccccactctgcaaacgcatgaaggtgcagatccgggactctgacaaggtcaatgatgtggccatcggcacc
3 CGCTATGGAATGAGCAGGTCGTCTTCACAGACTTGTTCCCCCCCACTCTGCAAACGCATGAAGGTGCAGATCCGGGACTCTGACAAGGTCAATGATGTGGCCATCGGCACC
4 cgctatggaatgagcaggtcgtcttcacagacttgttcccccccactctgcaaacgcatgaaggtgcagatccgggactctgacaaggtcaatgatgtggccatcggcacc
5 --------------------------------------------------------------------------------------------------------------
6 --------------------------------------------------------------------------------------------------------------
7 --------------------------------------------------------------------------------------------------------------
```

```
1550 1560 1570 1580 1590 1600 1610 1620 1630 1640 1650

NsbI
cacttcatcgacctgcgcaagatttccaacgatggagacaaaggcttcctgcctaccctcggtccagcctgggtgaacatgtacggctccacgcgcaactacacactgct
gtgaagtagctggacgcgttctaaaggttgctacctctgtttccgaaggacggatgggagccaggtcggacccacttgtacatgccgaggtgcgcgttgatgtgtgacga
Otoferlin CDS
PCR product
C2C
mExon 15

  cacttcatcgacctgcgcaagatttccaacgatggagacaaaggcttcctgcctaccctcggtccagcctgggtgaacatgtacggctccacgcgcaactacacactgct
1 cacttcatcgacctgcgcaagatttccaacgatggagacaaaggcttcctgcctaccctcggtccagcctgggtgaacatgtacggctccacgcgcaactacacactgct
2 cacttcatcgacctgcgcaagatttccaacgatggagacaaaggcttcctgcctaccctcggtccagcctgggtgaacatgtacggctccacgcgcaactacacactgct
3 CACTTCATCGACCTGCGCAAGATTTCCAACGATGGAGACAAAGGCTTCCTGCCTACCCTCGGTCCAGCCTGGGTGAACATGTACGGCTCCACGCGCAACTACACACTGCT
4 cacttcatcgacctgcgcaagatttccaacgatggagacaaaggcttcctgcctaccctcggtccagcctgggtgaacatgtacggctccacgcgcaactacacactgct
5 -------------------------------------------gcttcctgcctaccctcggtccagcctgggtgaacatgtacggctccacgcgcaactacacactgct
6 -------------------------------------------gcttcctgcctaccctcggtccagcctgggtgaacatgtacggctccacgcgcaactacacactgct
7 -------------------------------------------gcttcctgcctaccctcggtccagcctgggtgaacatgtacggctccacgcgcaactacacactgct
```

1660 1670 1680 1690 1700 1710 1720 1730 1740 1750 1760

ggacgagcaccaggacttgaatgaaggcctgggggagggtgtgtccttccgggcccgcctcatgttgggactagctgtggagatcctggacacctccaacccagagctca
cctgctcgtggtcctgaacttacttccggaccccctcccacacaggaaggcccgggcggagtacaaccctgatcgacacctctaggacctgtggaggttgggtctcgagt

Otoferlin CDS

PCR product

ggacgagcaccaggacttgaatgaaggcctgggggagggtgtgtccttccgggcccgcctcatgttgggactagctgtggagatcctggacacctccaacccagagctca
1 → ggacgagcaccaggacttgaatgaaggcctgggggagggtgtgtccttccgggcccgcctcatgttgggactagctgtggagatcctggacacctccaacccagagctca
2 → ggacgagcaccaggacttgaatgaaggcctgggggagggtgtgtccttccgggcccgcctcatgttgggactagctgtggagatcctggacacctccaacccagagctca
3 → GGACGAGCACCAGGACTTGAATGAAGGCCTGGGGGAGGGTGTGTCCTTCCGGGCCCGCCTCATGTTGGGACTAGCTGTGGAGATCCTGGACACCTCCAACCCAGAGCTCA
4 → ggacgagcaccaggacttgaatgaaggcctgggggagggtgtgtccttccgggcccgcctcatgttgggactagctgtggagatcctggacacctccaacccagagctca
5 → ggacgagcaccaggacttgaatgaaggcctgggggagggtgtgtccttccgggcccgcctcatgttgggactagctgtggagatcctggacacctccaacccagagctca
6 → ggacgagcaccaggacttgaatgaaggcctgggggagggtgtgtccttccgggcccgcctcatgttgggactagctgtggagatcctggacacctccaacccagagctca
7 → ggacgagcaccaggacttgaatgaaggcctgggggagggtgtgtccttccgggcccgcctcatgttgggactagctgtggagatcctggacacctccaacccagagctca

1770 1780 1790 1800 1810 1820 1830 1840 1850 1860 1870

ccagctccacggaggtgcaggtggagcaggccacgcctgtctcggagagctgcacagggagaatggaagaatttttctatttggagccttcttggaagcctcaatgatt
ggtcgaggtgcctccacgtccacctcgtccggtgcggacagagcctctcgacgtgtccctcttaccttcttaaaaagataaacctcggaagaaccttcggagttactaa

Otoferlin CDS

PCR product

ccagctccacggaggtgcaggtggagcaggccacgcctgtctcggagagctgcacagggagaatggaagaatttttctatttggagccttcttggaagcctcaatgatt
1 → ccagctccacggaggtgcaggtggagcaggccacgcctgtctcggagagctgcacagggagaatggaagaatttttctatttggagccttcttggaagcctcaatgatt
2 → ccagctccacggaggtgcaggtggagcaggccacgcctgtctcggagagctgcacagggagaatggaagaatttttctatttggagccttcttggaagcctcaatgatt
3 → CCAGCTCCACGGAGGTGCAGGTGGAGCAGGCCACGCCTGTCTCGGAGAGCTGCACAGGGAGAATGGAAGAATTTTTCTATTTGGAGCCTTCTTGGAAGCCTCAATGATT
4 → ccagctccacggaggtgcaggtggagcaggccacgcctgtctcggagagctgcacagggagaatggaagaatttttctatttggagccttcttggaagcctcaatgatt
5 → ccagctccacggaggtgcaggtggagcaggccacgcctgtctcggagagctgcacagggagaatggaagaatttttctatttggagccttcttggaagcctcaatgatt
6 → ccagctccacggaggtgcaggtggagcaggccacgcctgtctcggagagctgcacagggagaatggaagaatttttctatttggagccttcttggaagcctcaatgatt
7 → ccagctccacggaggtgcaggtggagcaggccacgcctgtctcggagagctgcacagggagaatggaagaatttttctatttggagccttcttggaagcctcaatgatt

1880 1890 1900 1910 1920 1930 1940 1950 1960 1970 1980

SfiI

gaccggaaaaatggggacaagccaattacctttgaggtgaccataggaaactacggcaatgaagtcgatggtatgtcccggcccctgaggcctcggccccggaaagagcc
ctggcctttttacccctgttcggttaatggaaactccactggtatcctttgatgccgttacttcagctaccatacagggccggggactccggagccggggcctttctcgg

Otoferlin CDS

PCR product

gaccggaaaaatggggacaagccaattacctttgaggtgaccataggaaactacggcaatgaagtcgatggtatgtcccggcccctgaggcctcggccccggaaagagcc
1 → gaccggaaaaatggggacaagccaattacctttgaggtgaccataggaaactacggcaatgaagtcgatggtatgtcccggcccctgaggcctcggccccggaaagagcc
2 → gaccggaaaaatggggacaagccaattacctttgaggtgaccataggaaactacggcaatgaagtcgatggtatgtcccggcccctgaggcctcggccccggaaagagcc
3 → GACCGGAAAAATGGGGACAAGCCAATTACCTTTGAGGTGACCATAGGAAACTACGGCAATGAAGTCGATGGTATGTCCCGGCCCCTGAGGCCTCGGCCCCGGAAAGAGCC
4 → gaccggaaaaatggggacaagccaattacctttgaggtgaccataggaaactacggcaatgaagtcgatggtatgtcccggcccctgaggcctcggccccggaaagagcc
5 → gaccggaaaaatggggacaagccaattacctttgaggtgaccataggaaactacggcaatgaagtcgatggtatgtcccggcccctgaggcctcggccccggaaagagcc
6 → gaccggaaaaatggggacaagccaattacctttgaggtgaccataggaaactacggcaatgaagtcgatggtatgtcccggcccctgaggcctcggccccggaaagagcc
7 → gaccggaaaaatggggacaagccaattacctttgaggtgaccataggaaactacggcaatgaagtcgatggtatgtcccggcccctgaggcctcggccccggaaagagcc

1990 2000 2010 2020 2030 2040 2050 2060 2070 2080 2090

tggggatgaagaagaggtagacctgattcagaactccagtgacgatgaaggtgacgaagccggggacctggcctcggtgtcctccacccccacctatgcggccccagatca
acccctacttcttctccatctggactaagtcttgaggtcactgctacttccactgcttcggcccctggaccggagccacaggaggtgggggtggatacgccggggtctagt

Otoferlin CDS

PCR product

tggggatgaagaagaggtagacctgattcagaactccagtgacgatgaaggtgacgaagccggggacctggcctcggtgtcctccacccccacctatgcggccccagatca
1 → tggggatgaagaagaggtagacctgattcagaactccagtgacgatgaaggtgacgaagccggggacctggcctcggtgtcctccacccccacctatgcggccccagatca
2 → tggggatgaagaagaggtagacctgattcagaactccagtgacgatgaaggtgacgaagccggggacctggcctcggtgtcctccacccccacctatgcggccccagatca
3 → TGGGGATGAAGAAGAGGTAGACCTGATTCAGAACTCCAGTGACGATGAAGGTGACGAAGCCGGGGACCTGGCCTCGGTGTCCTCCACCCCCACCTATGCGGCCCCAGATCA
4 → tggggatgaagaagaggtagacctgattcagaactccagtgacgatgaaggtgacgaagccggggacctggcctcggtgtcctccacccccacctatgcggccccagatca
5 → tggggatgaagaagaggtagacctgattcagaactccagtgacgatgaaggtgacgaagccggggacctggcctcggtgtcctccacccccacctatgcggccccagatca
6 → tggggatgaagaagaggtagacctgattcagaactccagtgacgatgaaggtgacgaagccggggacctggcctcggtgtcctccacccccacctatgcggccccagatca
7 → tggggatgaagaagaggtagacctgattcagaactccagtgacgatgaaggtgacgaagccggggacctggcctcggtgtcctccacccccacctatgcggccccagatca

2100 2110 2120 2130 2140 2150 2160 2170 2180 2190 2200

cggacaggaactatttccacctgccctacctggagcgcaagccctgcatctatatcaagagctggtggcctgaccagaggcggcgcctctacaatgccaacatcatggat
gcctgtccttgataaaggtggacgggatggacctcgcgttcgggacgtagatatagttctcgaccaccggactggtctccgccgcggagatgttacggttgtagtaccta

Otoferlin CDS

PCR product

cggacaggaactatttccacctgccctacctggagcgcaagccctgcatctatatcaagagctggtggcctgaccagaggcggcgcctctacaatgccaacatcatggat
1 → cggacaggaactatttccacctgccctacctggagcgcaagccctgcatctatatcaagagctggtggcctgaccagaggcggcgcctctacaatgccaacatcatggat
2 → cggacaggaactatttccacctgccctacctggagcgcaagccctgcatctatatcaagagctggtggcctgaccagaggcggcgcctctacaatgccaacatcatggat
3 → CGGACAGGAACTATTTCCACCTGCCCTACCTGGAGCGCAAGCCCTGCATCTATATCAAGAGCTGGTGGCCTGACCAGAGGCGGCGCCTCTACAATGCCAACATCATGGAT
4 → cggacaggaactatttccacctgccctacctggagcgcaagccctgcatctatatcaagagctggtggcctgaccagaggcggcgcctctacaatgccaacatcatggat
5 → cggacaggaactatttccacctgccctacctggagcgcaagccctgcatctatatcaagagctggtggcctgaccagaggcggcgcctctacaatgccaacatcatggat
6 → cggacaggaactatttccacctgccctacctggagcgcaagccctgcatctatatcaagagctggtggcctgaccagaggcggcgcctctacaatgccaacatcatggat
7 → cggacaggaactatttccacctgccctacctggagcgcaagccctgcatctatatcaagagctggtggcctgaccagaggcggcgcctctacaatgccaacatcatggat

2210 2220 2230 2240 2250 2260 2270 2280 2290 2300 2310

cacattgctgacaagctggaagaaggcctgaatgatgtacaggagatgatcaaaacggagaagtcctacccggagcgccgcctgcggggtgtgctagaggaactcagctg
gtgtaacgactgttcgaccttcttccggacttactacatgtcctctactagttttgcctcttcaggatgggcctcgcggcggacgccccacacgatctccttgagtcgac

Otoferlin CDS

PCR product

cacattgctgacaagctggaagaaggcctgaatgatgtacaggagatgatcaaaacggagaagtcctacccggagcgccgcctgcggggtgtgctagaggaactcagctg
1 → cacattgctgacaagctggaagaaggcctgaatgatgtacaggagatgatcaaaacggagaagtcctacccggagcgccgcctgcggggtgtgctagaggaactcagctg
2 → cacattgctgacaagctggaagaaggcctgaatgatgtacaggagatgatcaaaacggagaagtcctacccggagcgccgcctgcggggtgtgctagaggaactcagctg
3 → CACATTGCTGACAAGCTGGAAGAAGGCCTGAATGATGTACAGGAGATGATCAAAACGGAGAAGTCCTACCCGGAGCGCCGCCTGCGGGGTGTGCTAGAGGAACTCAGCTG
4 → cacattgctgacaagctggaagaaggcctgaatgatgtacaggagatgatcaaaacggagaagtcctacccggagcgccgcctgcggggtgtgctagaggaactcagctg
5 → cacattgctgacaagctggaagaaggcctgaatgatgtacaggagatgatcaaaacggagaagtcctacccggagcgccgcctgcggggtgtgctagaggaactcagctg
6 → cacattgctgacaagctggaagaaggcctgaatgatgtacaggagatgatcaaaacggagaagtcctacccggagcgccgcctgcggggtgtgctagaggaactcagctg
7 → cacattgctgacaagctggaagaaggcctgaatgatgtacaggagatgatcaaaacggagaagtcctacccggagcgccgcctgcggggtgtgctagaggaactcagctg

tggctgcca
accgacggt

Otoferlin CDS

PCR product

tggctgcca
1 → tggctgcca
2 → tggctgcca
3 → TGGCTGCCA
4 → tggctgcca
5 → tggctgcca
6 → tggctgccaGTGAGACTGATGGAGAGGGCAGGGTGGGACATGGGGACACTCTAGACAGGGCAGGGCAGAGGTGGCTAAGTCCTGGCTCTTCTACCCCTACCCCTCTGCAG
7 → tggctgcca

2330 2340 2350 2360 2370 2380 2390 2400 2410 2420

ccgcttcctctccctctcggacaaggaccagggccgctcgtcccgcaccaggctggatcgagagcgtcttaagtcctgtatgagggagttggagagcatgggacagcagg
ggcgaaggagagggagagcctgttcctggtcccggcgagcagggcgtggtccgacctagctctcgcagaattcaggacatactccctcaacctctcgtaccctgtcgtcc

Otoferlin CDS

PCR product

hExon 21

ccgcttcctctccctctcggacaaggaccagggccgctcgtcccgcaccaggctggatcgagagcgtcttaagtcctgtatgagggagttggagagcatgggacagcagg
1 → ccgcttcctctccctctcggacaaggaccagggccgctcgtcccgcaccaggctggatcgagagcgtcttaagtcctgtatgagggagttggagagcatgggacagcagg
2 → ccgcttcctctccctctcggacaaggaccagggccgctcgtcccgcaccaggctggatcgagagcgtcttaagtcctgtatgagggagttggagagcatgggacagcagg
3 → CCGCTTCCTCTCCCTCTCGGACAAGGACCAGGGCCGCTCGTCCCGCACCAGGCTGGATCGAGAGCGTCTTAAGTCCTGTATGAGGGAGTTGGAGAGCATGGGACAGCAGG
4 → ccgcttcctctccctctcggacaaggaccagggccgctcgtcccgcaccaggctggatcgagagcgtcttaagtcctgtatgagggagttggagagcatgggacagcagg
5 → ccgcttcctctccctctcTgacaaggaccagggccgctcgtcccgcaccaggctggatcgagagcgtcttaagtcctgtatgagggagCtggagagcatgggacagcagg
6 → ccgcttcctctccctctcTgacaaggaccagggccgctcgtcccgcaccaggctggatcgagagcgtcttaagtcctgtatgagggagCtggagagcatgggacagcagg
7 → ccgcttcctctccctctcTgacaaggaccagggccgctcgtcccgcaccaggctggatcgagagcgtcttaagtcctgtatgagggagCtggagagcatgggacagcagg

```
2440 2450 2460 2470 2480 2490 2500 2510 2520 2530
ccaagagcctgagggctcaggtgaagcggcacactgttcgggacaagctgaggtcatgccagaactttctgcagaagctacgcttcctggcggatgagccccagcacagc
ggttctcggactcccgagtccacttcgccgtgtgacaagccctgttcgactccagtacggtcttgaaagacgtcttcgatgcgaaggaccgcctactcggggtcgtgtcg
Otoferlin CDS
PCR product
hExon 21

  ccaagagcctgagggctcaggtgaagcggcacactgttcgggacaagctgaggtcatgccagaactttctgcagaagctacgcttcctggcggatgagccccagcacagc
1 ccaagagcctgagggctcaggtgaagcggcacactgttcgggacaagctgaggtcatgccagaactttctgcagaagctacgcttcctggcggatgagccccagcacagc
2 ccaagagcctgagggctcaggtgaagcggcacactgttcgggacaagctgaggtcatgccagaactttctgcagaagctacgcttcctggcggatgagccccagcacagc
3 CCAAGAGCCTGAGGGCTCAGGTGAAGCGGCACACTGTTCGGGACAAGCTGAGGTCATGCCAGAACTTTCTGCAGAAGCTACGCTTCCTGGCGGATGAGCCCCAGCACAGC
4 ccaagagcctgagggctcaggtgaagcggcacactgttcgggacaagctgaggtcatgccagaactttctgcagaagctacgcttcctggcggatgagccccagcacagc
5 ccaagagcctgagggctcaggtgaagcggcacactgttcgggacaagctgaggtTatgccagaactttctgcagaaActacgcttcctggcAgatgagccccagcacagc
6 ccaagagcctgagggctcaggtgaagcggcacactgttcgggacaagctgaggtTatgccagaactttctgcagaaActacgcttcctggcAgatgagccccagcacagc
7 ccaagagcctgagggctcaggtgaagcggcacactgttcgggacaagctgaggtTatgccagaactttctgcagaaActacgcttcctggcAgatgagccccagcacagc
```

```
2550 2560 2570 2580 2590 2600 2610 2620 2630 2640
Kpn2I
attccggatgtgttcatttggatgatgagcaacaacaaacgtatcgcctatgcccgcgtgccttccaaagacctgctcttctccatcgtggaggaggaactgggcaagga
taaggcctacacaagtaaacctactactcgttgttgtttgcatagcggatacgggcgcacggaaggtttctggacgagaagaggtagcacctcctccttgacccgttcct
Otoferlin CDS
PCR product
AccIII

  attccggatgtgttcatttggatgatgagcaacaacaaacgtatcgcctatgcccgcgtgccttccaaagacctgctcttctccatcgtggaggaggaactgggcaagga
1 attccggatgtgttcatttggatgatgagcaacaacaaacgtatcgcctatgcccgcgtgccttccaaagacctgctcttctccatcgtggaggaggaactgggcaagga
2 attccggatgtgttcatttggatgatgagcaacaacaaacgtatcgcctatgcccgcgtgccttccaaagacctgctcttctccatcgtggaggaggaactgggcaagga
3 ATTCCTGATGTGTTCATTTGGATGATGAGCAACAACAAACGTATCGCCTATGCCCGCGTGCCTTCCAAAGACCTGCTCTTCTCCATCGTGGAGGAGGAACTGGGCAAGGA
4 attccTgatgtgttcatttggatgatgagcaacaacaaacgtatcgcctatgcccgcgtgccttccaaagacctgctcttctccatcgtggaggaggaactgggcaagga
5 attccTgatgtgttcatttggatgatgagcaacaacaaacgtatcgcctatgcccgcgtgccttccaaagacctgctGttctccatcgtggaggaggaactgggcaagga
6 attccTgatgtgttcatttggatgatgagcaacaacaaacgtatcgcctatgcccgcgtgccttccaaagacctgctGttctccatcgtggaggaggaactgggcaagga
7 attccTgatgtgttcatttggatgatgagcaacaacaaacgtatcgcctatgcccgcgtgccttccaaagacctgctGttctccatcgtggaggaggaactgggcaagga
```

```
2660 2670 2680
ctgcgccaaagtcaagaccctcttcctgaag
gacgcggtttcagttctgggagaaggacttc
Otoferlin CDS
PCR product

  ctgcgccaaagtcaagaccctcttcctgaag
1 ctgcgccaaagtcaagaccctcttcctgaag
2 ctgcgccaaagtcaagaccctcttcctgaag
3 CTGCGCCAAAGTCAAGACCCTCTTCCTGAAG
4 ctgcgccaaagtcaagaccctcttcctgaag
5 ctgcgccaaagtcaagaccctcttcctgaagGTGCTGCGGGGGATGGGCCAAGCTCGAGTGGGTGGGGTGGGTTCCTGTAGCAACTCCGATGAGGCTGGCAAGCCTAAGA
6 ctgcgccaaagtcaagaccctcttcctgaag
7 ctgcgccaaagtcaagaccctcttcctgaag
```

```
1
2
3
4
5 GCAGGAGGTGGAGCCCTTAACTAGCAGGTGTTCAGGGGTCTGGAGCCCACCTGTCCCCACCTGCCTTCCCCCCTTAGTCTCTCATACCCAGCTCTGTCCGGTGAGGGGGT
6
7
```

```
1 →
2 →
3 →
4 →
5 → AGGCGAAGCTCTATCTACCTTTGCCTAAAGCTTCAACACCACCCTGGGCCTCCCTTACCCTGGGAGTCCTGGAAATTCTCAGGCTCTTGGTGACCCTGAGCCTGCCCCTA
6 →
7 →
```

```
      2690      2700      2710      2720      2730      2740      2750      2760      2770      2780
 ctgccagggaagaggggcttcggctcggcaggctggacagtacaggccaagctggagctctacctgtggctgggcctcagcaagcagcgaaaggacttcctgtgtggtc
 gacggtcccttctccccgaagccgagccgtccgacctgtcatgtccggttcgacctcgagatggacaccgacccggagtcgttcgtcgctttcctgaaggacacaccag
```

Otoferlin CDS

PCR product

```
      ctgccagggaagaggggcttcggctcggcaggctggacagtacaggccaagctggagctctacctgtggctgggcctcagcaagcagcgaaaggacttcctgtgtggtc
1 →   ctgccagggaagaggggcttcggctcggcaggctggacagtacaggccaagctggagctctacctgtggctgggcctcagcaagcagcgaaaggacttcctgtgtggtc
2 →   ctgccagggaagaggggcttcggctcggcaggctggacagtacaggccaagctggagctctacctgtggctgggcctcagcaagcagcgaaaggacttcctgtgtggtc
3 →   CTGCCAGGGAAGAGGGGCTTCGGCTCGGCAGGCTGGACAGTACAGGCCAAGCTGGAGCTCTACCTGTGGCTGGGCCTCAGCAAGCAGCGAAAGGACTTCCTGTGTGGTC
4 →   ctgccagggaagaggggcttcggctcggcaggctggacagtacaggccaagctggagctctacctgtggctgggcctcagcaagcagcgaaaggacttcctgtgtggtc
5 →  Gctgccagggaagaggggcttcggctcggcaggctggacagtacaggccaagctggagctctacctgtggctgggcctcagcaagcagcgaaaggacttcctgtgtggtc
6 →   ctgccagggaagaggggcttcggctcggcaggctggacagtacaggccaagctggagctctacctgtggctgggcctcagcaagcagcgaaaggacttcctgtgtggtc
7 →   ctgccagggaagaggggcttcggctcggcaggctggacagtacaggccaagctggagctctacctgtggctgggcctcagcaagcagcgaaaggacttcctgtgtggtc
```

```
      2800      2810      2820      2830      2840      2850      2860      2870      2880      2890
tgccctgtggcttcgaggaggtcaaggcagcccaaggcctgggcctgcattcctttccgcccatcagcctagtctacaccaagaagcaagccttccagctccgagcacac
acgggacaccgaagctcctccagttccgtcgggttccggacccggacgtaaggaaaggcgggtagtcggatcagatgtggttcttcgttcggaaggtcgaggctcgtgtg
```

Otoferlin CDS

PCR product

C2D

tcgtgtg

Rev Primer

```
     tgccctgtggcttcgaggaggtcaaggcagcccaaggcctgggcctgcattcctttccgcccatcagcctagtctacaccaagaagcaagccttccagctccgagcacac
1 →  tgccctgtggcttcgaggaggtcaaggcagcccaaggcctgggcctgcattcctttccgcccatcagcctagtctacaccaagaagcaagccttccagctccgagcacac
2 →  tgccctgtggcttcgaggaggtcaaggcagcccaaggcctgggcctgcattcctttccgcccatcagcctagtctacaccaagaagcaagccttccagctccgagcacac
3 →  TGCCCTGTGGCTTCGAGGAGGTCAAGGCAGCCCAAGGCCTGGGCCTGCATTCCTTTCCGCCCATCAGCCTAGTCTACACCAAGAAGCAAGCCTTCCAGCTCCGAGCACAC
4 →  tgccctgtggcttcgaggaggtcaaggcagcccaaggcctgggcctgcattcctttccgcccatcagcctagtctacaccaagaagcaagccttccagctccgagcacac
5 →  tgccctgtggcttcgaggaggtcaaggcagcccaaggcctgggcctgcattcctttccgcccatcagcctagtctacaccaagaagcaagccttccagctccgagcacac
6 →  tgccctgtggcttcgaggaggtcaaggcagcccaaggcctgggcctgcattcctttccgcccatcagcctagtctacaccaagaagcaagccttccagctccgagcacac
7 →  tgccctgtggcttcgaggaggtcaaggcagcccaaggcctgggcctgcattcctttccgcccatcagcctagtctacaccaagaagcaagccttccagctccgagcacac
```

```
      2910      2920      2930      2940      2950      2960      2970      2980      2990      3000
atgtatcaggcccgaagcctctttgctgctgacagcagtgggctctctgatccctttgcccgtgtcttcttcatcaaccagagccaatgcactgaggttctaaacgagac
tacatagtccgggcttcggagaaacgacgactgtcgtcacccgagagactagggaaacgggcacagaagaagtagttggtctcggttacgtgactccaagatttgctctg
```

Otoferlin CDS

PCR product

C2D

tacatagtccgggcttcggagaa

Rev Primer

```
     atgtatcaggcccgaagcctctttgctgctgacagcagtgggctctctgatccctttgcccgtgtcttcttcatcaaccagagccaatgcactgaggttctaaacgagac
1 →  atgtatcaggcccgaagcctctttgctgctgacagcagtgggctctctgatccctttgcccgtgtcttcttcatcaaccagagccaatgcactgaggttctaaacgagac
2 →  atgtatcaggcccgaagcctctt
3 →  ATGTATCAGGCCCGAAGCCTCTTTGCTGCTGACAGCAGTGGGCTCTCTGATCCCTTTGCCCGTGTCTTCTTCATCAACCAGAGCCAATGCACTGAGGTTCTAAACGAGAC
4 →  atgtatcaggcccgaagcctctt
5 →  atgtatcaggcccgaagcctctt
6 →  atgtatcaggcccgaagcctctt
7 →  atgtatcaggcccgaagcctctt
```

```
3020 3030 3040 3050 3060 3070 3080 3090 3100 3110
                                             BauI
actgtgtcccacctgggaccagatgctggtatttgacaacctggagctgtacggtgaagctcacgagttacgagatgatccccccatcattgtcattgaaatctacgacc
tgacacagggtggaccctggtctacgaccataaactgttggacctcgacatgccacttcgagtgctcaatgctctactaggggggtagtaacagtaactttagatgctgg
Otoferlin CDS
C2D

  actgtgtcccacctgggaccagatgctggtatttgacaacctggagctgtacggtgaagctcacgagttacgagatgatccccccatcattgtcattgaaatctacgacc
1 actgtgtcccacctgggaccagatgctggtatttgacaacctggagctgtacggtgaagctcacgagttacgagatgatccccccatcattgtcattgaaatctacgacc
3 ACTGTGTCCCACCTGGGACCAGATGCTGGTATTTGACAACCTGGAGCTGTACGGTGAAGCTCACGAGTTACGAGATGATCCCCCCATCATTGTCATTGAAATCTACGACC

3130 3140 3150 3160 3170 3180 3190 3200 3210 3220
aggacagcatgggcaaagccgacttcatgggccggaccttcgccaagcccctggtgaagatggcagatgaagcatactgcccacctcgcttcccgccgcagcttgagtac
tcctgtcgtacccgtttcggctgaagtacccggcctggaagcggttcggggaccacttctaccgtctacttcgtatgacgggtggagcgaagggcggcgtcgaactcatg
Otoferlin CDS
C2D

  aggacagcatgggcaaagccgacttcatgggccggaccttcgccaagcccctggtgaagatggcagatgaagcatactgcccacctcgcttcccgccgcagcttgagtac
1 aggacagcatgggcaaagccgacttcatgggccggaccttcgccaagcccctggtgaagatggcagatgaagcatactgcccacctcgcttcccgccgcagcttgagtac
3 AGGACAGCATGGGCAAAGCCGACTTCATGGGCCGGACCTTCGCCAAGCCCCTGGTGAAGATGGCAGATGAAGCATACTGCCCACCTCGCTTCCCGCCGCAGCTTGAGTAC

3240 3250 3260 3270 3280 3290 3300 3310 3320 3330
taccagatctaccgaggcagtgccactgccggagacctactggctgccttcgagctgctgcagattgggccatcagggaaggctgacctgccacccatcaatggcccagt
atggtctagatggctccgtcacggtgacggcctctggatgaccgacggaagctcgacgacgtctaacccggtagtcccttccgactggacggtgggtagttaccgggtca
Otoferlin CDS
C2D

  taccagatctaccgaggcagtgccactgccggagacctactggctgccttcgagctgctgcagattgggccatcagggaaggctgacctgccacccatcaatggcccagt
1 taccagatctaccgaggcagtgccactgccggagacctactggctgccttcgagctgctgcagattgggccatcagggaaggctgacctgccacccatcaatggcccagt
3 TACCAGATCTACCGAGGCAGTGCCACTGCCGGAGACCTACTGGCTGCCTTCGAGCTGCTGCAGATTGGGCCATCAGGGAAGGCTGACCTGCCACCCATCAATGGCCCAGT

3350 3360 3370 3380 3390 3400 3410 3420 3430 3440
ggacatggacagagggcccatcatgcctgtgcccgtgggaatccggccagtgctcagcaagtaccgagtggaggtgctgttctggggcctgagggacctaaagagggtga
cctgtacctgtctcccgggtagtacggacacgggcacccttaggccggtcacgagtcgttcatggctcacctccacgacaagaccccggactccctggatttctcccact
Otoferlin CDS

  ggacatggacagagggcccatcatgcctgtgcccgtgggaatccggccagtgctcagcaagtaccgagtggaggtgctgttctggggcctgagggacctaaagagggtga
1 ggacatggacagagggcccatcatgcctgtgcccgtgggaatccggccagtgctcagcaagtaccgagtggaggtgctgttctggggcctgagggacctaaagagggtga
3 GGACATGGACAGAGGGCCCATCATGCCTGTGCCCGTGGGAATCCGGCCAGTGCTCAGCAAGTACCGAGTGGAGGTGCTGTTCTGGGGCCTGAGGGACCTAAAGAGGGTGA

3460 3470 3480 3490 3500 3510 3520 3530 3540 3550
                                                                AanI
acctggcccaggtggaccgaccacgggtggacatcgagtgtgcaggaaaggggggtacaatcctccctgattcacaattataagaagaaccccaacttcaacacgctggtc
tggaccgggtccacctggctggtgcccacctgtagctcacacgtcctttccccccatgttaggagggactaagtgttaatattcttcttggggttgaagttgtgcgaccag
Otoferlin CDS

  acctggcccaggtggaccgaccacgggtggacatcgagtgtgcaggaaaggggggtacaatcctccctgattcacaattataagaagaaccccaacttcaacacgctggtc
1 acctggcccaggtggaccgaccacgggtggacatcgagtgtgcaggaaaggggggtacaatcctccctgattcacaattataagaagaaccccaacttcaacacgctggtc
3 ACCTGGCCCAGGTGGACCGACCACGGGTGGACATCGAGTGTGCAGGAAAGGGGGGTACAATCCTCCCTGATTCACAATTATAAGAAGAACCCCAACTTCAACACGCTGGTC

3570 3580 3590 3600 3610 3620 3630 3640 3650 3660
aagtggtttgaagtggacctcccggagaatgagctcctgcacccacccttgaacatccgagtggtagattgccgggcctttggacgatacaccctggtgggttcccacgc
ttcaccaaacttcacctggagggcctcttactcgaggacgtgggtgggaacttgtaggctcaccatctaacggcccggaaacctgctatgtgggaccacccaagggtgcg
Otoferlin CDS

  aagtggtttgaagtggacctcccggagaatgagctcctgcacccacccttgaacatccgagtggtagattgccgggcctttggacgatacaccctggtgggttcccacgc
1 aagtggtttgaagtggacctcccggagaatgagctcctgcacccacccttgaacatccgagtggtagattgccgggcctttggacgatacaccctggtgggttcccacgc
3 AAGTGGTTTGAAGTGGACCTCCCGGAGAATGAGCTCCTGCACCCACCCTTGAACATCCGAGTGGTAGATTGCCGGGCCTTTGGACGATACACCCTGGTGGGTTCCCACGC

3680 3690 3700 3710 3720 3730 3740 3750 3760 3770
agtcagctcactgaggcgcttcatctaccgacctccagaccgctcagccccccaactggaacaccacaggggaggttgtagtaagcatggagcctgaggagccagttaaga
tcagtcgagtgactccgcgaagtagatggctggaggtctggcgagtcggggggttgaccttgtggtgtcccctccaacatcattcgtacctcggactcctcggtcaattct
Otoferlin CDS

  agtcagctcactgaggcgcttcatctaccgacctccagaccgctcagccccccaactggaacaccacaggggaggttgtagtaagcatggagcctgaggagccagttaaga
1 agtcagctcactgaggcgcttcatctaccgacctccagaccgctcagccccccaactggaacaccacaggggaggttgtagtaagcatggagcctgaggagccagttaaga
3 AGTCAGCTCACTGAGGCGCTTCATCTACCGACCTCCAGACCGCTCAGCCCCCCAACTGGAACACCACAGGGGAGGTTGTAGTAAGCATGGAGCCTGAGGAGCCAGTTAAGA
```

3790 3800 3810 3820 3830 3840 3850 3860 3870 3880

agctggagaccatggtgaaactggatgcgacttctgatgctgtggtcaaggtggatgtggctgaagatgagaaggaaaggaagaagaagaaaaagaaaggcccgtcagag
tcgacctctggtaccactttgacctacgctgaagactacgacaccagttccacctacaccgacttctactcttcctttccttcttcttctttttctttccgggcagtctc

Otoferlin CDS

agctggagaccatggtgaaactggatgcgacttctgatgctgtggtcaaggtggatgtggctgaagatgagaaggaaaggaagaagaagaaaaagaaaggcccgtcagag
1 → agctggagaccatggtgaaactggatgcgacttctgatgctgtggtcaaggtggatgtggctgaagatgagaaggaaaggaagaagaagaaaaagaaaggcccgtcagag
3 → AGCTGGAGACCATGGTGAAACTGGATGCGACTTCTGATGCTGTGGTCAAGGTGGATGTGGCTGAAGATGAGAAGGAAAGGAAGAAGAAGAAAAAGAAAGGCCCGTCAGAG

3900 3910 3920 3930 3940 3950 3960 3970 3980 3990

PaeI

gagccagaggaggaagagcccgatgagagcatgctggattggtggtccaagtacttcgcctccatcgacacaatgaaggagcaacttcgacaacatgagacctctggaac
ctcggtctcctccttctcgggctactctcgtacgacctaaccaccaggttcatgaagcggaggtagctgtgttacttcctcgttgaagctgttgtactctggagaccttg

Otoferlin CDS

gagccagaggaggaagagcccgatgagagcatgctggattggtggtccaagtacttcgcctccatcgacacaatgaaggagcaacttcgacaacatgagacctctggaac
1 → gagccagaggaggaagagcccgatgagagcatgctggattggtggtccaagtacttcgcctccatcgacacaatgaaggagcaacttcgacaacatgagacctctggaac
3 → GAGCCAGAGGAGGAAGAGCCCGATGAGAGCATGCTGGATTGGTGGTCCAAGTACTTCGCCTCCATCGACACAATGAAGGAGCAACTTCGACAACATGAGACCTCTGGAAC

4010 4020 4030 4040 4050 4060 4070 4080 4090 4100

Eco47III

tgacttggaagagaaggaagagatggaaagcgctgagggcctgaagggaccaatgaagagcaaggagaagtccagagctgcaaaggaggagaaaaagaagaaaaaccaga
actgaaccttctcttccttctctacctttcgcgactcccggacttccctggttacttctcgttcctcttcaggtctcgacgtttcctcctctttttcttctttttggtct

Otoferlin CDS

tgacttggaagagaaggaagagatggaaagcgctgagggcctgaagggaccaatgaagagcaaggagaagtccagagctgcaaaggaggagaaaaagaagaaaaaccaga
1 → tgacttggaagagaaggaagagatggaaagcgctgagggcctgaagggaccaatgaagagcaaggagaagtccagagctgcaaaggaggagaaaaagaagaaaaaccaga
3 → TGACTTGGAAGAGAAGGAAGAGATGGAAAGCGCTGAGGGCCTGAAGGGACCAATGAAGAGCAAGGAGAAGTCCAGAGCTGCAAAGGAGGAGAAAAAGAAGAAAAACCAGA

4120 4130 4140 4150 4160 4170 4180 4190 4200 4210

Bsu15I*

gccctggccctggccagggatcggaggctcctgagaagaagaaagccaagatcgatgagcttaaggtgtaccccaaggagctggaatcggagtttgacagctttgaggac
cgggaccgggaccggtccctagcctccgaggactcttcttctttcggttctagctactcgaattccacatggggttcctcgaccttagcctcaaactgtcgaaactcctg

Otoferlin CDS

gccctggccctggccagggatcggaggctcctgagaagaagaaagccaagatcgatgagcttaaggtgtaccccaaggagctggaatcggagtttgacagctttgaggac
1 → gccctggccctggccagggatcggaggctcctgagaagaagaaagccaagatcgatgagcttaaggtgtaccccaaggagctggaatcggagtttgacagctttgaggac
3 → GCCCTGGCCCTGGCCAGGGATCGGAGGCTCCTGAGAAGAAGAAAGCCAAGATCGATGAGCTTAAGGTGTACCCCAAGGAGCTGGAATCGGAGTTTGACAGCTTTGAGGAC

4230 4240 4250 4260 4270 4280 4290 4300 4310 4320

tggctgcacaccttcaacctgttgaggggcaagacgggagatgatgaggatggctccacagaggaggagcgcatagtaggccgattcaagggctccctctgtgtgtacaa
accgacgtgtggaagttggacaactccccgttctgccctctactactcctaccgaggtgtctcctcctcgcgtatcatccggctaagttcccgagggagacacacatgtt

Otoferlin CDS

tggctgcacaccttcaacctgttgaggggcaagacgggagatgatgaggatggctccacagaggaggagcgcatagtaggccgattcaagggctccctctgtgtgtacaa
1 → tggctgcacaccttcaacctgttgaggggcaagacgggagatgatgaggatggctccacagaggaggagcgcatagtaggccgattcaagggctccctctgtgtgtacaa
3 → TGGCTGCACACCTTCAACCTGTTGAGGGGCAAGACGGGAGATGATGAGGATGGCTCCACAGAGGAGGAGCGCATAGTAGGCCGATTCAAGGGCTCCCTCTGTGTGTACAA

4340 4350 4360 4370 4380 4390 4400 4410 4420 4430

XhoI

agtgccactcccagaagatgtatctcgagaagctggctatgatcccacctatggaatgttccagggcatcccaagcaatgaccccatcaatgtgctggtccgaatctatg
tcacggtgagggtcttctacatagagctcttcgaccgatactagggtggataccttacaaggtcccgtagggttcgttactggggtagttacacgaccaggcttagatac

Otoferlin CDS

C2E

agtgccactcccagaagatgtatctcgagaagctggctatgatcccacctatggaatgttccagggcatcccaagcaatgaccccatcaatgtgctggtccgaatctatg
1 → agtgccactcccagaagatgtatctcgagaagctggctatgatcccacctatggaatgttccagggcatcccaagcaatgaccccatcaatgtgctggtccgaatctatg
3 → AGTGCCACTCCCAGAAGATGTATCTCGAGAAGCTGGCTATGATCCCACCTATGGAATGTTCCAGGGCATCCCAAGCAATGACCCCATCAATGTGCTGGTCCGAATCTATG

4450 4460 4470 4480 4490 4500 4510 4520 4530 4540

tggtccgggccacagacctgcacccggccgacatcaatggcaaagctgacccctatattgccatcaagttaggcaagaccgacatccgagacaaggagaactacatctcc
accaggcccggtgtctggacgtgggccggctgtagttaccgtttcgactggggatataacggtagttcaatccgttctggctgtaggctctgttcctcttgatgtagagg

Otoferlin CDS

C2E

tggtccgggccacagacctgcacccggccgacatcaatggcaaagctgacccctatattgccatcaagttaggcaagaccgacatccgagacaaggagaactacatctcc
1 → tggtccgggccacagacctgcacccggccgacatcaatggcaaagctgacccctatattgccatcaagttaggcaagaccgacatccgagacaaggagaactacatctcc
3 → TGGTCCGGGCCACAGACCTGCACCCGGCCGACATCAATGGCAAAGCTGACCCCTATATTGCCATCAAGTTAGGCAAGACCGACATCCGAGACAAGGAGAACTACATCTCC

4560 4570 4580 4590 4600 4610 4620 4630 4640 4650

aagcagctcaaccctgtgtttgggaagtcctttgacattgaggcctccttccccatggagtccatgttgacagtggccgtgtacgactgggatctggtgggcactgatga

ttcgtcgagttgggacacaaacccttcaggaaactgtaactccggaggaaggggtacctcaggtacaactgtcaccggcacatgctgaccctagaccacccgtgactact

Otoferlin CDS

C2E

aagcagctcaaccctgtgtttgggaagtcctttgacattgaggcctccttccccatggagtccatgttgacagtggccgtgtacgactgggatctggtgggcactgatga

1 → aagcagctcaaccctgtgtttgggaagtcctttgacattgaggcctccttccccatggagtccatgttgacagtggccgtgtacgactgggatctggtgggcactgatga

3 → AAGCAGCTCAACCCTGTGTTTGGGAAGTCCTTTGACATTGAGGCCTCCTTCCCCATGGAGTCCATGTTGACAGTGGCCGTGTACGACTGGGATCTGGTGGGCACTGATGA

4670 4680 4690 4700 4710 4720 4730 4740 4750 4760

KflI

cctcatcggagaaaccaagattgacctggaaaaccgcttctacagcaagcatcgcgccacctgcggcatcgcacagacctattccatacatggctacaatatctggaggg

ggagtagcctctttggttctaactggaccttttggcgaagatgtcgttcgtagcgcggtggacgccgtagcgtgtctggataaggtatgtaccgatgttatagacctccc

Otoferlin CDS

C2E

cctcatcggagaaaccaagattgacctggaaaaccgcttctacagcaagcatcgcgccacctgcggcatcgcacagacctattccatacatggctacaatatctggaggg

1 → cctcatcggagaaaccaagattgacctggaaaaccgcttctacagcaagcatcgcgccacctgcggcatcgcacagacctattccatacatggctacaatatctggaggg

3 → CCTCATCGGAGAAACCAAGATTGACCTGGAAAACCGCTTCTACAGCAAGCATCGCGCCACCTGCGGCATCGCACAGACCTATTCCATACATGGCTACAATATCTGGAGGG

4780 4790 4800 4810 4820 4830 4840 4850 4860 4870

accccatgaagcccagccagatcctgacacgcctctgtaaagagggcaaagtggacggcccccactttggtccccatgggagagtgagggttgccaaccgtgtcttcacg

tggggtacttcgggtcggtctaggactgtgcggagacatttctcccgtttcacctgccgggggtgaaaccaggggtaccctctcactcccaacggttggcacagaagtgc

Otoferlin CDS

C2E

accccatgaagcccagccagatcctgacacgcctctgtaaagagggcaaagtggacggcccccactttggtccccatgggagagtgagggttgccaaccgtgtcttcacg

1 → accccatgaagcccagccagatcctgacacgcctctgtaaagagggcaaagtggacggcccccactttggtccccatgggagagtgagggttgccaaccgtgtcttcacg

3 → ACCCCATGAAGCCCAGCCAGATCCTGACACGCCTCTGTAAAGAGGGCAAAGTGGACGGCCCCCACTTTGGTCCCCATGGGAGAGTGAGGGTTGCCAACCGTGTCTTCACG

4890 4900 4910 4920 4930 4940 4950 4960 4970 4980

gggccttcagaaatagaggatgagaatggtcagaggaagcccacagatgagcacgtggcactgtctgctctgagacactgggaggacatcccccgggtgggctgccgcct

cccggaagtctttatctcctactcttaccagtctccttcgggtgtctactcgtgcaccgtgacagacgagactctgtgaccctcctgtagggggcccacccgacggcgga

Otoferlin CDS

gggccttcagaaatagaggatgagaatggtcagaggaagcccacagatgagcacgtggcactgtctgctctgagacactgggaggacatcccccgggtgggctgccgcct

1 → gggccttcagaaatagaggatgagaatggtcagaggaagcccacagatgagcacgtggcactgtctgctctgagacactgggaggacatcccccgggtgggctgccgcct

3 → GGGCCTTCAGAAATAGAGGATGAGAATGGTCAGAGGAAGCCCACAGATGAGCACGTGGCACTGTCTGCTCTGAGACACTGGGAGGACATCCCCCGGGTGGGCTGCCGCCT

5000 5010 5020 5030 5040 5050 5060 5070 5080 5090

tgtgccggaacacgtggagaccaggccgctgctcaaccctgacaagccaggcattgagcagggccgcctggagctgtgggtggacatgttccccatggacatgccagccc

acacggccttgtgcacctctggtccggcgacgagttgggactgttcggtccgtaactcgtcccggcggacctcgacacccacctgtacaaggggtacctgtacggtcggg

Otoferlin CDS

tgtgccggaacacgtggagaccaggccgctgctcaaccctgacaagccaggcattgagcagggccgcctggagctgtgggtggacatgttccccatggacatgccagccc

1 → tgtgccggaacacgtggagaccaggccgctgctcaaccctgacaagccaggcattgagcagggccgcctggagctgtgggtggacatgttccccatggacatgccagccc

3 → TGTGCCGGAACACGTGGAGACCAGGCCGCTGCTCAACCCTGACAAGCCAGGCATTGAGCAGGGCCGCCTGGAGCTGTGGGTGGACATGTTCCCCATGGACATGCCAGCCC

5110 5120 5130 5140 5150 5160 5170 5180 5190 5200

ctgggacacctctggatatatcccccaggaaacccaagaagtacgagctgcgggtcatcgtgtggaacacagacgaggtggtcctggaagacgatgatttcttcacggga

gaccctgtggagacctatatagggggtcctttgggttcttcatgctcgacgcccagtagcacaccttgtgtctgctccaccaggaccttctgctactaaagaagtgccct

Otoferlin CDS

C2F

ctgggacacctctggatatatcccccaggaaacccaagaagtacgagctgcgggtcatcgtgtggaacacagacgaggtggtcctggaagacgatgatttcttcacggga

1 → ctgggacacctctggatatatcccccaggaaacccaagaagtacgagctgcgggtcatcgtgtggaacacagacgaggtggtcctggaagacgatgatttcttcacggga

3 → CTGGGACACCTCTGGATATATCCCCCAGGAAACCCAAGAAGTACGAGCTGCGGGTCATCGTGTGGAACACAGACGAGGTGGTCCTGGAAGACGATGATTTCTTCACGGGA

5220 5230 5240 5250 5260 5270 5280 5290 5300 5310

gagaagtccagtgacatttttgtgaggggggtggctgaagggccagcaggaggacaaacaggacacagatgtccactatcactccctcacgggggagggcaacttcaactg

ctcttcaggtcactgtaaaaacactccccccaccgacttcccggtcgtcctcctgtttgtcctgtgtctacaggtgatagtgagggagtgccccctcccgttgaagttgac

Otoferlin CDS

C2F

gagaagtccagtgacatttttgtgaggggggtggctgaagggccagcaggaggacaaacaggacacagatgtccactatcactccctcacgggggagggcaacttcaactg

1 → gagaagtccagtgacatttttgtgaggggggtggctgaagggccagcaggaggacaaacaggacacagatgtccactatcactccctcacgggggagggcaacttcaactg

3 → GAGAAGTCCAGTGACATTTTTGTGAGGGGGGTGGCTGAAGGGCCAGCAGGAGGACAAACAGGACACAGATGTCCACTATCACTCCCTCACGGGGGAGGGCAACTTCAACTG

5330 5340 5350 5360 5370 5380 5390 5400 5410 5420

gagatacctcttccccttcgactacctagcggccgaagagaagatcgttatgtccaaaaaggagtctatgttctcctgggatgagacggagtacaagatccctgcgcggc
ctctatggagaaggggaagctgatggatcgccggcttctcttctagcaatacaggtttttcctcagatacaagaggaccctactctgcctcatgttctagggacgcgccg

Otoferlin CDS

C2F

gagatacctcttccccttcgactacctagcggccgaagagaagatcgttatgtccaaaaaggagtctatgttctcctgggatgagacggagtacaagatccctgcgcggc
1 gagatacctcttccccttcgactacctagcggccgaagagaagatcgttatgtccaaaaaggagtctatgttctcctgggatgagacggagtacaagatccctgcgcggc
3 GAGATACCTCTTCCCCTTCGACTACCTAGCGGCCGAAGAGAAGATCGTTATGTCCAAAAAGGAGTCTATGTTCTCCTGGGATGAGACGGAGTACAAGATCCCTGCGCGGC

5440 5450 5460 5470 5480 5490 5500 5510 5520 5530

BshTI **Alw44I**

tcaccctgcagatctgggacgctgaccacttctcggctgacgacttcctgggggctatcgagctggacctgaaccggttcccgaggggcgctaagacagccaagcagtgc
agtgggacgtctagaccctgcgactggtgaagagccgactgctgaaggacccccgatagctcgacctggacttggccaagggctccccgcgattctgtcggttcgtcacg

Otoferlin CDS

C2F

tcaccctgcagatctgggacgctgaccacttctcggctgacgacttcctgggggctatcgagctggacctgaaccggttcccgaggggcgctaagacagccaagcagtgc
1 tcaccctgcagatctgggacgctgaccacttctcggctgacgacttcctgggggctatcgagctggacctgaaccggttcccgaggggcgctaagacagccaagcagtgc
3 TCACCCTGCAGATCTGGGACGCTGACCACTTCTCGGCTGACGACTTCCTGGGGGCTATCGAGCTGGACCTGAACCGGTTCCCGAGGGGCGCTAAGACAGCCAAGCAGTGC

5550 5560 5570 5580 5590 5600 5610 5620 5630 5640

DraI

accatggagatggccaccggggaggtggacgtacccctggtttccatctttaaacagaaacgtgtcaaaggctggtggcccctcctggcccgcaatgagaatgatgagtt
tggtacctctaccggtggcccctccacctgcatggggaccaaaggtagaaatttgtctttgcacagtttccgaccaccggggaggaccgggcgttactcttactactcaa

Otoferlin CDS

C2F

accatggagatggccaccggggaggtggacgtacccctggtttccatctttaaacagaaacgtgtcaaaggctggtggcccctcctggcccgcaatgagaatgatgagtt
1 accatggagatggccaccggggaggtggacgtacccctggtttccatctttaaacagaaacgtgtcaaaggctggtggcccctcctggcccgcaatgagaatgatgagtt
3 ACCATGGAGATGGCCACCGGGGAGGTGGACGTACCCCTGGTTTCCATCTTTAAACAGAAACGTGTCAAAGGCTGGTGGCCCCTCCTGGCCCGCAATGAGAATGATGAGTT

5660 5670 5680 5690 5700 5710 5720 5730 5740 5750

tgagctcacaggcaaagtggaggcggagctacacctactcacggcagaggaggcagagaagaaccctgtgggcctggctcgcaatgaacctgatcccctagaaaaaccca
actcgagtgtccgtttcacctccgcctcgatgtggatgagtgccgtctcctccgtctcttcttgggacacccggaccgagcgttacttggactaggggatctttttgggt

Otoferlin CDS

tgagctcacaggcaaagtggaggcggagctacacctactcacggcagaggaggcagagaagaaccctgtgggcctggctcgcaatgaacctgatcccctagaaaaaccca
1 tgagctcacaggcaaagtggaggcggagctacacctactcacggcagaggaggcagagaagaaccctgtgggcctggctcgcaatgaacctgatcccctagaaaaaccca
3 TGAGCTCACAGGCAAAGTGGAGGCGGAGCTACACCTACTCACGGCAGAGGAGGCAGAGAAGAACCCTGTGGGCCTGGCTCGCAATGAACCTGATCCCCTAGAAAAACCCA

5770 5780 5790 5800 5810 5820 5830 5840 5850 5860

accggcctgacacggcattcgtctggttcctgaacccactcaaatctatcaagtacctcatctgcacccggtacaagtggctgatcatcaagatcgtgctggcgctgctg
tggccggactgtgccgtaagcagaccaaggacttgggtgagtttagatagttcatggagtagacgtgggccatgttcaccgactagtagttctagcacgaccgcgacgac

Otoferlin CDS

accggcctgacacggcattcgtctggttcctgaacccactcaaatctatcaagtacctcatctgcacccggtacaagtggctgatcatcaagatcgtgctggcgctgctg
1 accggcctgacacggcattcgtctggttcctgaacccactcaaatctatcaagtacctcatctgcacccggtacaagtggctgatcatcaagatcgtgctggcgctgctg
3 ACCGGCCTGACACGGCATTCGTCTGGTTCCTGAACCCACTCAAATCTATCAAGTACCTCATCTGCACCCGGTACAAGTGGCTGATCATCAAGATCGTGCTGGCGCTGCTG

5880 5890 5900 5910 5920 5930 5940

XmaJI ***End*** (5942)

gggctgctcatgctggccctcttcctttacagcctcccaggctacatggtcaagaagctcctaggggcctgag 3′
cccgacgagtacgaccgggagaaggaaatgtcggagggtccgatgtaccagttcttcgaggatccccggactc 5′

Otoferlin CDS

gggctgctcatgctggccctcttcctttacagcctcccaggctacatggtcaagaagctcctaggggcctgag
1 gggctgctcatgctggccctcttcctttacagcctcccaggctacatggtcaagaagctcctaggggcctgag
3 GGGCTGCTCATGCTGGCCCTCTTCCTTTACAGCCTCCCAGGCTACATGGTCAAGAAGCTCCTAGGGGCCTGA

Original Sequence:

1: DualAAV mOtof CDS
5942 bases
1 .. 5942

2: (*) DualAAV-TS (1753 bp)
1753 bases
1 .. 1753

3: WT mOtof var4 CDS (NM_001313767.1)
5934 bases
1 .. 5934 (2 mismatches)

4: (*) WT (1753 bp)
1753 bases
1 .. 1753 (1 mismatch)

5: (c) Otof-/- (1679 bp)
1679 bases
1 .. 1679 (7 mismatches, 2 gaps)

6: (b) Otof-/- (1480 bp)
1480 bases
1 .. 1480 (7 mismatches, 2 gaps)

7: (a) Otof-/- (1379 bp)
1379 bases
1 .. 1379 (7 mismatches, 1 gap)
