# Supplementary material for: A dual‐AAV approach restores fast exocytosis and partially rescues auditory function in deaf otoferlin knock‐out mice
Source: EMBO Mol Med. 2018 Dec 3;11(1):e9396. doi: 10.15252/emmm.201809396 (PMC6328916; doi:10.15252/emmm.201809396)

Source data for figure EV4B
EtBr stained agarose gel

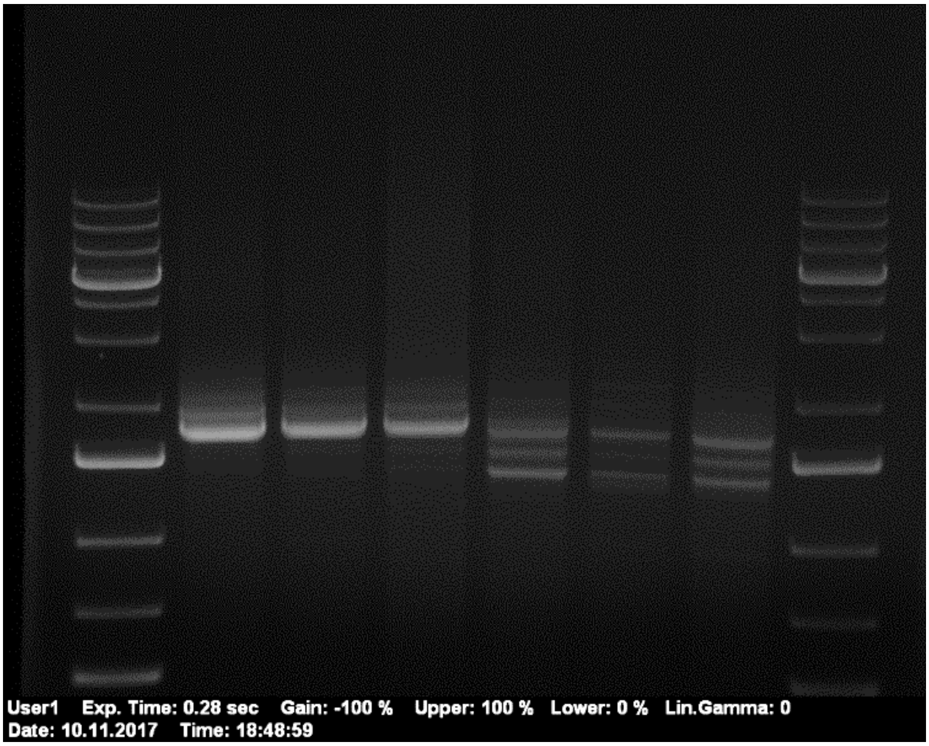

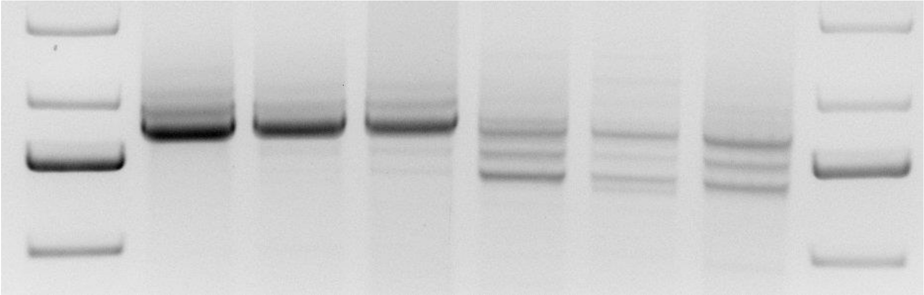

Source Data for Figure EV4D

Mouse anti otoferlin antibody (Abcam)

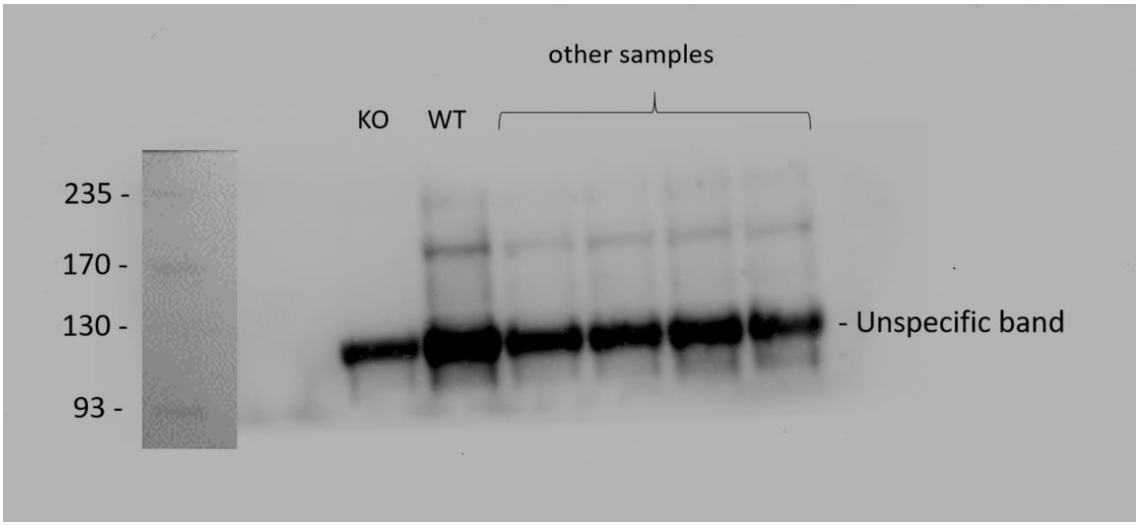

Rabbit anti GAPDH antibody (SySy)

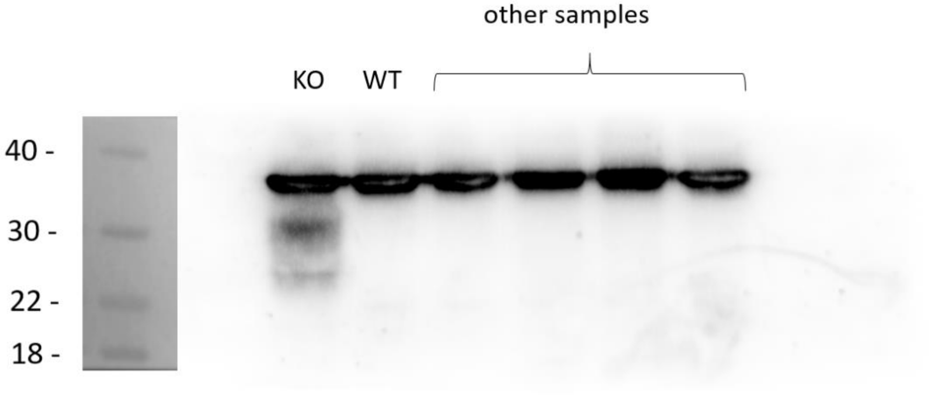

Supplement: Supplementary file 3 — Source Data for Expanded View [file EMMM-11-e9396-s003.zip › Fig_EV4_source_data/EMM-2018-09396-V3-SourceData-FigEV4BD.pdf]
